# Marriage of Virus‐Mimic Surface Topology and Microbubble‐Assisted Ultrasound for Enhanced Intratumor Accumulation and Improved Cancer Theranostics

**DOI:** 10.1002/advs.202004670

**Published:** 2021-05-14

**Authors:** Zheying Meng, Yang Zhang, E Shen, Wei Li, Yanjie Wang, Krishnan Sathiyamoorthy, Wei Gao, Michael C. Kolios, Wenkun Bai, Bing Hu, Wenxing Wang, Yuanyi Zheng

**Affiliations:** ^1^ Department of Ultrasound in Medicine Shanghai Jiao Tong University Affiliated Sixth People's Hospital Shanghai 200233 P. R. China; ^2^ Shanghai Institute of Ultrasound in Medicine Shanghai Jiao Tong University Affiliated Sixth People's Hospital Shanghai 200233 P. R. China; ^3^ Department of Chemistry Shanghai Key Laboratory of Molecular Catalysis and Innovative Materials Fudan University Shanghai 200433 P. R. China; ^4^ Department of Physics Ryerson University Toronto Ontario M5B 2K3 Canada; ^5^ Department of Ultrasound in Medicine Shanghai Jiao Tong University Affiliated Sixth People's Hospital, Institute of Medical Imaging Shanghai Jiao Tong University Shanghai 200233 P. R. China; ^6^ Department of Ultrasound in Medicine Shanghai Jiao Tong University Affiliated Sixth People's Hospital State Key Laboratory of Oncogenes and Related Genes Shanghai Jiao Tong University School of Medicine Shanghai 200032 P. R. China

**Keywords:** cancer theranostics, intratumor accumulation, mesoporous silica, microbubble‐assisted ultrasound, multimodal theranostic, virus‐mimic surface topology

## Abstract

The low delivery efficiency of nanoparticles to solid tumors greatly reduces the therapeutic efficacy and safety which is closely related to low permeability and poor distribution at tumor sites. In this work, an “intrinsic plus extrinsic superiority” administration strategy is proposed to dramatically enhance the mean delivery efficiency of nanoparticles in prostate cancer to 6.84% of injected dose, compared to 1.42% as the maximum in prostate cancer in the previously reported study. Specifically, the intrinsic superiority refers to the virus‐mimic surface topology of the nanoparticles for enhanced nano–bio interactions. Meanwhile, the extrinsic stimuli of microbubble‐assisted low‐frequency ultrasound is to enhance permeability of biological barriers and improve intratumor distribution. The enhanced intratumor enrichment can be verified by photoacoustic resonance imaging, fluorescence imaging, and magnetic resonance imaging in this multifunctional nanoplatform, which also facilitates excellent anticancer effect of photothermal treatment, photodynamic treatment, and sonodynamic treatment via combined laser and ultrasound irradiation. This study confirms the significant advance in nanoparticle accumulation in multiple tumor models, which provides an innovative delivery paradigm to improve intratumor accumulation of nanotherapeutics.

## Graphical abstract

This work proposes the combination of intrinsic optimization the nanostructure as virus‐mimic surface topology to enhance nano–bio interactions and extrinsic stimuli of microbubble‐assisted low‐frequency ultrasound to vastly improve intratumor permeability and distribution of nanoparticles in multiple cancer models. The multifunctional nanoplatform is capable with trimodal imaging to determine the optimal therapeutic timing, which demonstrates a successful combined anticancer effect.

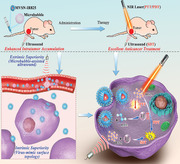

## 1 Introduction

The low delivery efficiency (DE) of nanoparticles into solid tumors leading to reduced therapeutic efficacy and aggravated side effects is the key factor that hinders the clinical translation of cancer nanomedicine, which is closely related to low permeability and poor distribution at tumor sites.^[^ ^1^, ^2^, ^3^ ^]^ For a long time, the enhanced permeability and retention effect has been regarded as the preferential theoretical mechanism for the passive retention of nanoparticles in solid tumors.^[^ ^4^ *^–^* ^6^ ^]^ Recently, a thought‐provoking study demonstrated that the transportation of nanoparticles from blood vessels into solid tumors was an active process occurring via a transendothelial mechanism rather than passive extravasation through interendothelial gaps.^[^ ^7^ ^]^ In either case, the efforts to increase the DE of nanoparticles into solid tumors never cease.^[^ ^8^, ^9^ ^]^ Generally, current valuable targeted designs of nanoparticles included modification of the physiochemical properties,^[^ ^10^ ^]^ decoration with active targeting ligands,^[^ ^11^ ^]^ elaborately designed stimuli‐responsive nanocarriers,^[^ ^12^ ^]^ and biomimetic coating with cell membranes,^[^ ^13^ ^]^ which could relatively extend the blood circulation and improve tumor uptake to a certain degree.^[^ ^14^ ^]^ Yet, the complicated and time‐consuming manufacture procedure, the obstacles of ligands‐installed nanocarriers have limited their clinical translation.^[^ ^15^ ^]^ In addition, the latest meta‐analysis manifested that there wass no statistically significant improvement in tumor delivery efficiency of nanoparticles between the studies before and after 2015,^[^ ^2^ ^]^ which suggested the necessity to explore a new delivery strategy for nanotherapeutics.

Microbubble‐assisted ultrasound (MAUS) with the superiority of noninvasive, safe, and frequently used in clinics is able to enhance the membrane permeability temporarily to allow therapeutics passing through biological barriers, such as vascular endothelium and cytomembrane, ^[^ ^16^, ^17^ ^]^ which utilizes acoustic cavitation produced by ultrasound exciting microbubbles. The possible mechanisms under cellular level have been studied including transient pore formation on cell membrane,^[^ ^18^, ^19^ ^]^ enhanced endocytosis,^[^ ^20^, ^21^ ^]^ and sonoprinting.^[^ ^22^ ^]^ To the best of our knowledge, this passive targeted delivery strategy has been validated as favorable for enhancing the targeted delivery of genes or drugs in cancer,^[^ ^23^, ^24^ ^]^ and other numerous disease models,^[^ ^25^ ^]^ such as stroke,^[^ ^26^ ^]^ and cardiac disease.^[^ ^27^ ^]^ Nonetheless, while the mean delivery efficiency of nanoparticles themselves, as gene or drug carriers, was super low up to 1.42% of injected dose as the maximum in prostate cancer,^[^ ^2^, ^3^ ^]^ which was also the key knotty issue in cancer nanomedicine.^[^ ^3^, ^7^ ^]^ In this study, we used low‐frequency 500 kHz ultrasound, instead of 1 MHz ultrasound that were mostly employed by most current studies.^[^ ^15^ ^]^ As we all know, lower frequency ultrasound has the advantage of higher tissue penetration, which could further promote the delivery of nanoparticles in deep tumor. Additionally, lower frequency ultrasound with lower acoustic pressure threshold could more efficiently trigger collapse cavitation than higher frequency ultrasound,^[^ ^28^, ^29^ ^]^ which facilitated to improve the membrane permeability temporarily. Therefore, microbubble‐assisted low‐frequency ultrasound is a delivery paradigm worth exploring to promote the intratumor accumulation of nanoparticles.^[^ ^30^ ^]^

From the perspective of the intrinsic nature of nanoparticles, surface topology could deeply affect the nanoscale extravasational competence when nanoparticles have a similar surface area, charge, and surface coating.^[^ ^31^, ^32^ ^]^ Nanoscale surface roughness could greatly increase surface area for nano–bio interactions and minimize repulsive interactions (for example, electrostatic, hydrophilic), thereby promoting adhesion, which might translate into easier engulfment by cells.^[^ ^33^, ^34^, ^35^ ^]^ Thus, we assumed the nanoparticles with nanoscale viral rough surface topology could enable favorable nano–bio interaction. Additionally, our previous work confirmed that virus‐mimic surface structured nanoparticles entered into cancer cells with a higher speed than the smooth and the mesoporous structured nanoparticles.^[^ ^35^ ^]^ Unfortunately, further study in vivo was not conducted at that time. Therefore, this work further verified the in vivo impact of virus‐mimic surface structure in comparison with the smooth and the mesoporous structure. To our knowledge, a study to explore the impacts of virus‐mimic surface topology combined with microbubble‐assisted low‐frequency ultrasound on both cellular uptake performance and intratumor enrichment has not been reported before.

**Figure 1:**
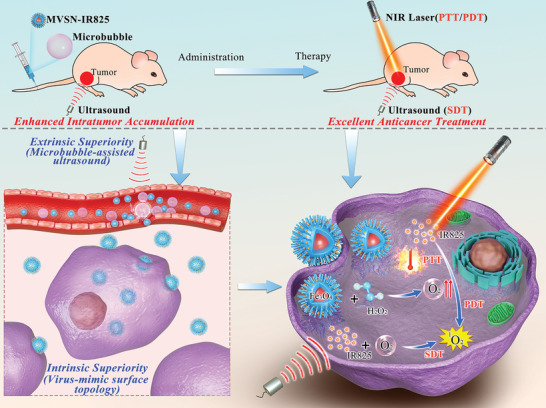
Schematic illustration. The virus‐mimic surface topological nanoparticles MVSN‐IR825 and clinical ultrasound microbubbles SonoVue were fully mixed and slowly injected into the tail vein. Meanwhile, low‐frequency ultrasound irradiation was targeted on tumors. The marriage of intrinsic virus‐mimic surface topology superiority and the extrinsic stimulation of ultrasound can significantly improve intratumor accumulation of nanoparticles. IR825 served as the PAI, PTT, and FI agent and Fe_3_O_4_ was used as a T2 contrast agent in MRI, which is indispensable in the clinical diagnosis of cancer; moreover, Fe_3_O_4_ could react with excess H_2_O_2_ in the tumor microenvironment to produce oxygen to facilitate the production of ROS in PDT and SDT. Low‐frequency ultrasound was used to irradiate the tumor site both during the intravenous administration for enhancing intratumor accumulation of nanoparticles and during the therapeutic procedure for SDT.

Herein, an “intrinsic plus extrinsic superiority” strategy was developed to systematically demonstrate that marriage of virus‐mimic surface topology and microbubble‐assisted low‐frequency ultrasound can significantly increase the penetration and distribution of nanoparticles in tumors. To monitor enhanced intratumor accumulation of nanoparticles, our exquisitely designed core–shell nanoplatform was capable of trimodal photoacoustic/fluorescence/magnetic resonance imaging (PAI/FI/MRI) due to IR825 conjugating the magnetic virus‐mimic surface topological mesoporous silica Fe_3_O_4_@vSiO_2_ (abbreviated as MVSN‐IR825), which provided the best timing of trimodal photothermal/photodynamic/sonodynamic treatment (PTT/PDT/SDT) with combined laser and ultrasound irradiation and demonstrated superior therapeutic efficacy. In detail, the commercial dye molecule IR825 was used for PAI, PTT, and FI agent on the strength of its near‐infrared (NIR) absorbance character. Notably, we discovered that IR825 could also work as sonosensitizer. Fe_3_O_4_ was used as T2 contrast agent of MRI, which was indispensable in clinical diagnosis of cancer. Additionally, Fe_3_O_4_ could react with excess H_2_O_2_ in the tumor microenvironment to produce oxygen to facilitate the production of reactive oxygen species (ROS) in PDT and SDT.^[^ ^36^ *^–^* ^38^ ^]^ Low‐frequency ultrasound was used to irradiate the tumor site both during intravenous administration for target delivery strategy and in the therapeutic procedure for SDT (**Figure** **1**). Considering that the cancer type significantly affects the intratumor accumulation of nanoparticles, with DE being particularly low for prostate cancer.^[^ ^2^, ^3^ ^]^ Thus, improving the intratumor accumulation of nanoparticles in prostate cancer is of great significance for advanced prostate cancer patients who are intolerant to surgery or hormone‐independent unsuitable for endocrine therapy.^[^ ^39^ ^]^ In this work, we mainly used PC‐3 xenograft tumors for detailed exploration. To further confirm the effectiveness of our strategy, we performed in vitro AGS gastric cancer cells and 143B osteosarcoma cells, and in vivo PC‐3 orthotopic tumors and 143B xenograft tumors for further verification. This work clearly demonstrated that the “intrinsic plus extrinsic superiority” strategy dramatically improved the intratumor penetration and distribution of nanoparticles for enhanced cancer theranostics.

## 2 Results and Discussion

### 2.1 Synthesis and Characterization of Nanoparticles

**Figure 2:**
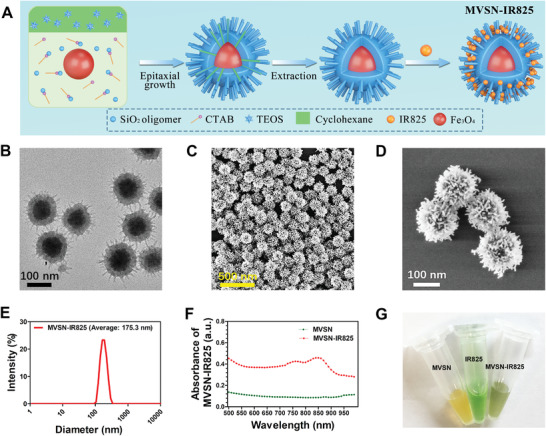
Fabrication and characterization of the MVSN‐IR825. A) Illustration of synthesis procedure of MVSN‐IR825. B) TEM image of MVSN‐IR825. C,D) SEM images of MVSN‐IR825. E) Size distribution of MVSN‐IR825. F) UV–vis–NIR absorbance spectra of the MVSN‐IR825 (1 mg mL^−1^) and MVSN (1 mg mL^−1^) in aqueous solution. G) Photograph of MVSN, IR825, and MVSN‐IR825.

A schematic illustration of the brief MVSN‐IR825 formation was presented in **Figure** **2**A. Briefly, MVSNs were fabricated by the single‐micelle epitaxial growth method according to our previous study.^[^ ^36^ ^]^ To improve the biocompatibility, the surface of the MVSNs were modified with polyethylene glycol (PEG) (Figures S1–S3, Supporting Information), which facilitated to the subsequent connection with fluorescein isothiocyanate (FITC) (Figure S4, Supporting Information) and IR825 (Figure S5A, Supporting Information). Notably, IR825 was recognized as a PTT agent with excellent photostability and photothermal conversion and a PAI/FI agent with high spatial resolution and deep tissue penetration.^[^ ^40^, ^41^, ^42^ ^]^ Recently, its role as a photosensitizer was also revealed,^[^ ^43^ ^]^ and other NIR heptamethine dyes, such as IR780, were reported to work as sonosensitizers.^[^ ^44^ ^]^ Thus, IR825 was expected to build a multifunctional nanoplatform with simple manufacturing requirements.^[^ ^45^ ^]^ To improve the biocompatibility of nanoparticles and avoid phagocytosis by mononuclear phagocyte system, we used PEG as surface‐coating of the nanoparticles.^[^ ^46^ ^]^ As revealed by transmission electron microscopy (TEM) and scanning electron microscopy (SEM) imaging, MVSN‐IR825 showed an obvious virus‐mimic surface topology with a uniform size of ≈160 nm, which was composed of two parts: spherical magnetic Fe_3_O_4_ core with a diameter of ≈80 nm and virus‐mimic silica shell formed by the vertical silica nanospikes with a length of ≈20 nm and a diameter of ≈7 nm (Figure 2B–E, Supporting Information). Energy‐dispersive X‐ray (EDX) spectrometry proved the presence of Fe, Si, and O elements (Figure S5C, Supporting Information), which were in uniform distribution exhibited by EDX elemental mapping, and the rough topology with vertical silica spiny nanotubes mimicking the surface glycoprotein spike of virus were clearly exhibited with high angle annular dark field scanning transmission electron microscopy (HAADF‐STEM) (Figure S5D, Supporting Information). The obtained MVSN‐IR825 dispersed well in water and ultraviolet (UV)– visable (vis)–NIR spectra exhibited a broad absorption in the near‐infrared region with a characteristic peak at ≈850 nm (Figure 2F,G). The conjugation rate (CR%) of MVSN‐IR825 was ≈66.7% and the drug loading (DL%) was about 1.6% (Figure S6, Supporting Information). Meanwhile, the core–shell structured nanocomposites with magnetic Fe_3_O_4_ cores and smooth/mesoporous silica shells were prepared for control samples, which were further conjugated with IR825 via the same method and abbreviated as MSSN‐IR825 and MMSN‐IR825 (the ER%/DL% of MSSN‐IR825 and MMSN‐IR825 was ≈70.8%/1.7% and 87.5%/2.1%, respectively.

### 2.2 In Vitro Study of Cell Endocytosis

**Figure 3:**
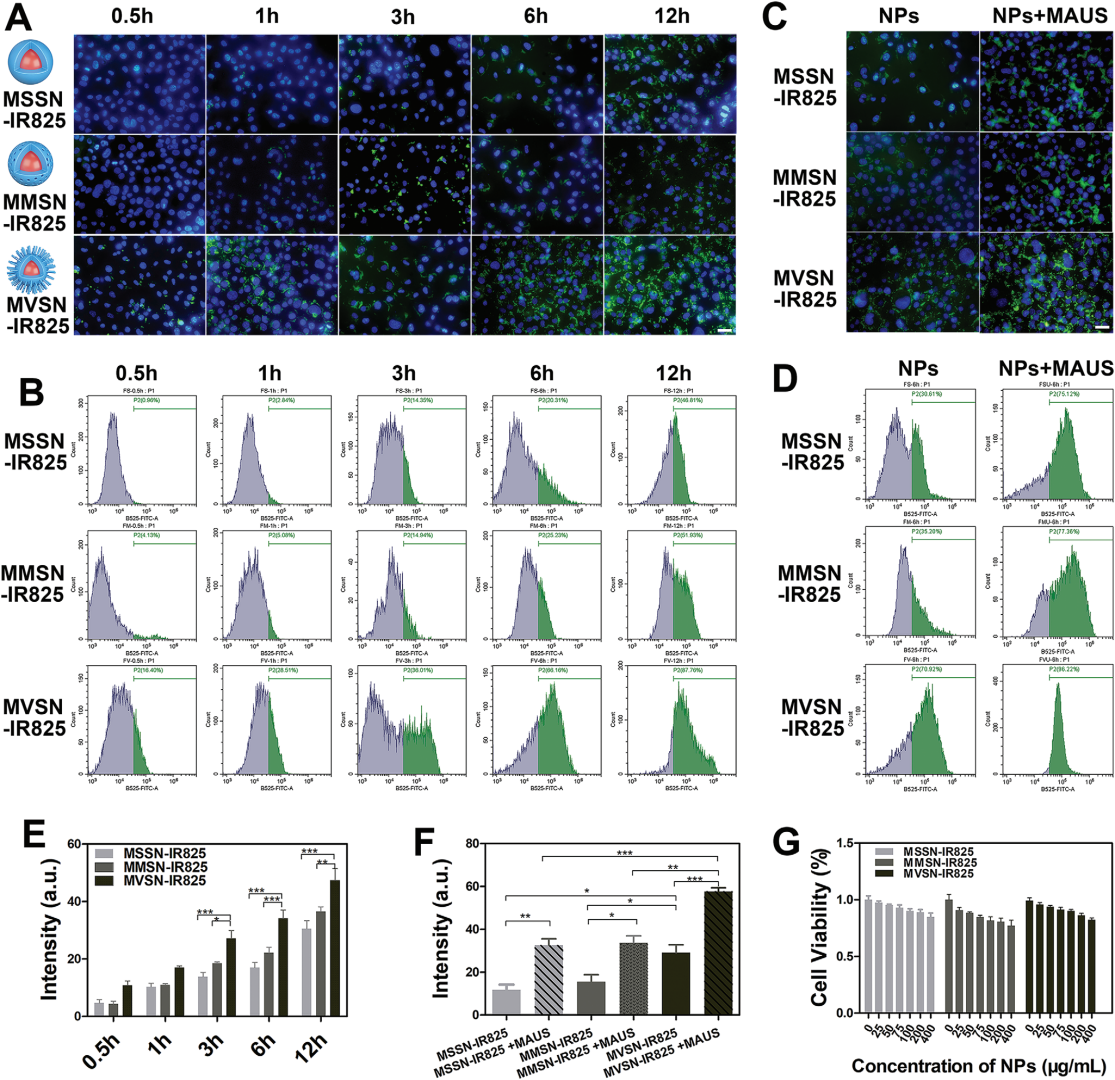
In vitro study on PC‐3 cell endocytosis. A) Fluorescence imaging and B) flow cytometry demonstrating cell phagocytosis of nanoparticles with different surface topology. C) Fluorescence imaging and D) flow cytometry showing effect of microbubble‐ assisted ultrasound on cell phagocytosis of nanoparticles. E,F) ImageJ pixel counting analysis of green fluorescence signal in fluorescence microscopy image (A) and (C), respectively. (E: *n* = 3 per group, mean ± SEM, two‐way analysis of variance (ANOVA), Bonferroni post‐test; F: *n* = 3 per group, mean ± SEM, one‐way ANOVA, Tukey post‐test. *P < 0.05, **P < 0.01, ***P < 0.001.) G) Relative viabilities of PC‐3 cells after incubation with FITC‐labeled MSSN‐IR825, MMSN‐IR825, MVSN‐IR825 at different concentrations for 24 h (*n* = 6 per group, mean ± SEM, two‐way ANOVA, Bonferroni post‐test. No significant difference, *P* > 0.5) (Scale: 50 µm).

To explore the effect of surface topology on cell phagocytosis, FITC‐labeled MSSN‐IR825/MMSN‐IR825/MVSN‐IR825 were incubated together with PC‐3 cells for different time periods and 4,6‐diamidino‐2‐phenylindole (DAPI) staining of cell nuclei was used to display intracellular phagocytosis of nanoparticles in fluorescence microscopy imaging. The MVSN‐IR825 group exhibited much stronger intracellular green fluorescence than MSSN‐IR825 and MMSN‐IR825 in same incubation period (**Figure** **3**A). Additionally, quantitative analysis of the mean green fluorescence intensity of PC‐3 cells by ImageJ pixel counting plugin also proved the same result (Figure 3E). Apart from this, another quantitative analysis of flow cytometry examination (Figure 3B) also showed that the MVSN‐IR825 exhibited more efficient cellular internalization than MSSN‐IR825 and MMSN‐IR825, which could be attributed to the enhanced nano–bio interactions induced by the virus‐mimic rough surface.

Apart from the intrinsic surface topology of the nanoparticles, we further studied the effect of extrinsic ultrasound stimulation on the cell endocytosis. In ultrasound stimulation groups, the three nanoparticles fully mixed with clinical ultrasound contrast microbubbles SonoVue were added into PC‐3 cells and irradiated by the low‐frequency ultrasound, then incubated for 6 h. Fluorescence microscopy images demonstrated more rapid internalization of all three nanoparticles with the help of ultrasound stimulation (Figure 3C). In particular, MVSN‐IR825 with ultrasound stimulation exhibited the highest cellular uptake efficiency in semiquantitative analysis of FITC intensity by fluorescence microscopy imaging and flow cytometry (Figure 3F,D). In addition, further cellular verification was conducted by using other two cancer cells: 143B osteosarcoma cells and AGS gastric cancer cells. After 6 h of incubation with different topographical nanoparticles and the stimulation of MAUS, the strongest green FITC fluorescent signal that measured by fluorescence microscopy imaging and flow cytometry analysis were appeared in group MVSN‐IR825 + MAUS in both 143B cells (Figure S7C,E, Supporting Information) and AGS cells (Figure S7D,F, Supporting Information). Thus, the combination of intrinsic morphological advantage and the external microbubble‐assisted ultrasound advantage was confirmed to be effective on the phagocytosis of nanoparticles at the cellular level. Next, a standard cell counting Kit‐8 (CCK‐8) assay was applied to characterize the cytotoxicity of the three nanoparticles, which showed a low cytotoxicity even at concentrations of 400 µg mL^−1^ after 24 h of incubation (Figure 3G). In addition, the MVSN‐IR825 showed a longer blood circulation time than that of MSSN‐IR825 and MMSN‐IR825 (Figure S8, Supporting Information), which was consistent with the previous reports.^[^ ^36^ ^]^

### 2.3 In Vivo Study on Intratumor Accumulation of Nanoparticles

**Figure 4:**
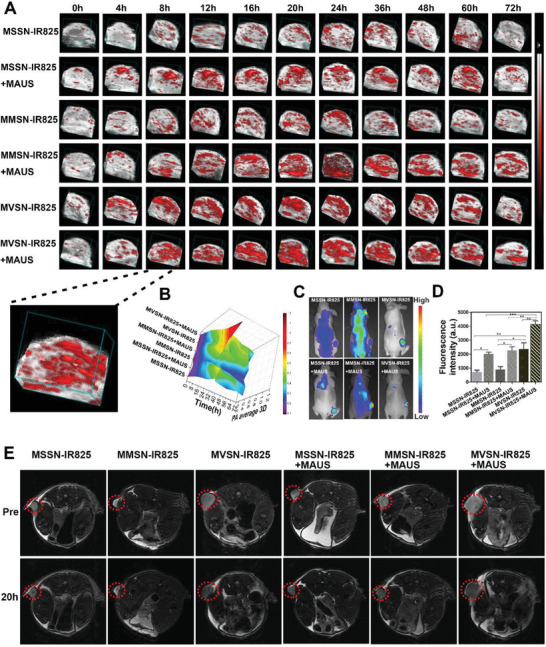
Multiple imaging methods monitoring the enrichment of nanoparticles in PC‐3 xenograft tumor‐bearing mice. A) 3D‐PAI monitoring the photoacoustic signal in tumors (the dotted line refers to the enlarged image clearly showing the distribution of nanoparticles in 3D tumor). B) Changes of 3D‐PA signal in per unit tumor volume over time in each group. C) In vivo fluorescence imaging monitoring of nanoparticles enrichment in tumor after 20 h of administration in each group. D) Quantitative analysis of fluorescence intensity per unit tumor area (*n* = 3 per group, mean ± SEM, one‐way ANOVA, Tukey post‐test. *P < 0.05, **P < 0.01, ***P < 0.001). E) MRI showing T2 signal changes of the tumor in each group before and 20 h after administration.

Encouraged by the results of cell endocytosis, we further validate the effect of “intrinsic and extrinsic superiority” on intratumor accumulation in vivo. First, hematoxylin and eosin (H&E) staining was carried out to detect toxicity of major organs 24 h after the intravenous injection of all three nanoparticles at concentration of 60 mg kg^−1^, which indicated good biocompatibility (Figure S9, Supporting Information). Then, human prostate cancer cell line PC‐3 was used to establish the xenograft heterotopic model owing to the potential of integrating optical diagnosis and treatment together in clinic.^[^ ^47^ ^]^ The tumor‐bearing mice were divided into the following six groups: MSSN‐IR825, MMSN‐IR825, MVSN‐IR825, MSSN‐IR825 + MAUS (microbubble‐assisted low‐frequency ultrasound delivery strategy was abbreviated for “MAUS” in experiment of intratumor accumulation), MMSN‐IR825 + MAUS, MVSN‐IR825 + MAUS. Then, at different time period after administration, the real‐time dynamic intratumor accumulation of nanoparticles was monitored by PAI, which was profited by the NIR absorbance of IR825. Specially, 3D‐PAI was chosen to display the spatial distribution of nanoparticles inside tumors, which was more objective and visualized than a single section of 2D‐PAI. As shown in **Figure** **4**A, the intratumor photoacoustic (PA) signal of the MVSN‐IR825 increased most significantly with the peak accumulation at 20 h, which was much stronger than that of the MSSN‐IR825 and MMSN‐IR825. When using microbubble‐assisted ultrasound delivery strategy, the intratumor photoacoustic signals were significantly enhanced and the MVSN‐IR825 + MAUS group showed the strongest intratumor PA signal at each time point. Quantitative 3D‐PA signal intensity per unit volume of tumors clearly demonstrated that the maximum enrichment inside the tumor was 20 h after injection in MVSN‐IR828 + MAUS group, which could be used as the optimal time point for subsequent treatment to ensure the maximum therapeutic effect (Figure 4B). In addition to PAI, FI in vivo was also used to record the intratumor enrichment of nanoparticles 20 h after intravenous administration. Similarly, the MVSN‐IR825 + MAUS group exhibited the highest fluorescence intensity compared to the other five groups (Figure 4C,D). Furthermore, as a revolutionary medical imaging in clinical diagnosis of prostate cancer, MRI was also used to evaluate the intratumor accumulation of nanoparticles because Fe_3_O_4_ contained in MVSN‐IR825 was an excellent T2 contrast (Figure S11, Supporting Information). Significant darkened T2‐contrast effect in tumor site could be clearly displayed 20 h after injection of nanoparticles, and that of MVSN‐IR825 + MAUS group was the most significant (Figure 4E). The above imaging results not only reflected the technological success of enhanced intratumor accumulation by using the marriage of virus‐mimic surface topology and microbubble‐assisted ultrasound, but also confirmed the multimodal imaging capability of MVSN‐IR825.

**Figure 5:**
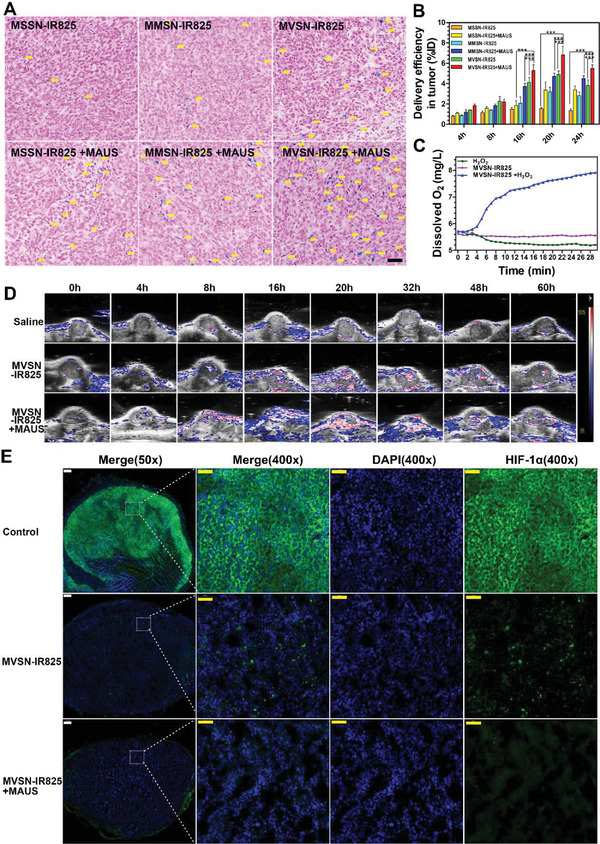
Improved intratumor accumulation assisted to alleviate tumor hypoxia. A) Prussian blue staining showing iron distribution in PC‐3 xenograft tumor‐bearing mice (Scale: 50 µm). B) ICP detecting the Fe content in tumors of mice in each group at different times. (Three mice were used for each group and each sample was measured with two repeats, mean ± SEM, two‐way ANOVA, Bonferroni post‐test. ***P < 0.001.) C) Dissolved oxygen meter detecting oxygen concentration of MVSN mixed with H_2_O_2_. D) PAI showing oxygen saturation of tumor in each group. E) HIF‐1*α* staining of tumor tissue (The short white scale: 200 µm, the long yellow scale: 50 µm).

Next, Prussian blue staining was further used to observe iron deposition inside the tumor. The result clearly showed the most significant increase in iron deposition could be observed in MVSN‐IR825 + MAUS group, which had been transferred into the deep stroma outside from the tumor vessels (**Figure** **5**A). Additionally, quantitative inductively coupled plasma (ICP) analysis of the amounts of accumulated nanoparticles showed that with marriage of the intrinsic virus‐mimic surface topology and extrinsic irradiation of tumors with microbubble‐assisted low‐frequency ultrasound, the mean delivery efficiency in PC‐3 xenograft tumor model was dramatically improved to 6.84% of injection dose (%ID) in MVSN‐IR825 + MAUS group (Figure 5B). Compared with reported studies on delivery efficiency of nanoparticles to prostate cancer using xenograft heterotopic models,^[^ ^2^, ^3^ ^]^ our results exhibited relatively obvious progress of intratumor accumulation of nanoparticles, which was obviously surpassed with the maximum value 1.42% of prostate cancer in previous report.^[^ ^2^ ^]^ Higher nonspecific uptake of the nanoparticles by spleen and liver was also observed in all groups, but the level of liver uptake and spleen uptake in group MVSN‐IR825 + MAUS were almost the lowest (Figure S12, Supporting Information).

To further confirm the efficacy of our “intrinsic and extrinsic superiority,” the PC‐3 orthotopic tumor model was employed because the prostate anatomically held much richer microcirculation than that of surrounding organs, which was an ideal goal for ultrasound targeting externally. At 20 h intravenous administration, in vivo FI showed that there was more fluorescence intensity of nanoparticles in MVSN‐IR825 group than MSSN‐IR825 and MMSN‐IR825 group, and the highest fluorescence intensity appeared in the MVSN‐IR825 + MAUS group (Figure S13A,B, Supporting Information). In addition, MRI images also exhibited the most decreased T2 signal of the orthotopic tumor in the MVSN‐IR825 + MAUS group (Figure S13C, Supporting Information), which further manifested the feasibility of our strategy to improve the intratumor accumulation of nanoparticles. Moreover, Prussian blue staining reflecting iron deposition inside the orthotopic tumor also showed the most significant accumulation of iron in deep stroma outside from the tumor vessels in MVSN‐IR825 + MAUS group (Figure S13D, Supporting Information).

Next, we employed subcutaneous xenograft osteosarcoma model constructed by luciferase labeled human 143B cell line to further confirm the efficacy of the “intrinsic and extrinsic superiority” strategy. FI in vivo 20 h after intravenous administration in each group clearly showed the highest fluorescence intensity in MVSN‐IR825 group, which suggested our strategy also apply to the osteosarcoma model (Figure S14A,B, Supporting Information). Thus, the combination of intrinsic morphological advantage and the external microbubble‐assisted ultrasound advantage was verified to improve the intratumor accumulation of nanoparticles by xenograft tumor models and the orthotopic model in vivo.

### 2.4 Improvement of Hypoxic State in Tumor

In general, iron oxide can act like a catalase with the ability of converting H_2_O_2_ to H_2_O and O_2_ to alleviate the hypoxic state inside the tumor,^[^ ^48^ ^]^ which is an important predecessor for PDT and SDT. To evaluate the enzyme‐like catalysis activity of MVSN‐IR825, the O_2_ production at different time period were detected. As shown in Figure 5C, the concentration of dissolved oxygen gradually increased in 30 min. To further explore oxygen production in vivo, the oxygen saturation of PC‐3 xenograft tumor‐bearing mice were assessed by PAI. With the enrichment of nanoparticles in tumors, the hypoxia state in tumors was greatly improved (Figure 5D). Quantitative analysis results showed that the oxygen saturation in the tumors reached the highest level at 20 h postinjection, and the intratumor oxygen content of MVSN‐IR825 + MAUS group was higher than that of MVSN‐IR825 group (Figure S15, Supporting Information), which was attributed to the effective enrichment of nanoparticles in the MVSN‐IR825 + MAUS group. In particular, the hypoxia‐inducible factor‐1*α* (HIF1‐*α*) immunofluorescence of tumors was performed, in which green fluorescence intensity of HIF‐1*α* was significantly high in the control group due to the hypoxia environment inside tumor (Figure 5E). The green fluorescence intensity significantly decreased with intratumor accumulation of MVSN‐IR825, while the lowest HIF‐1*α* content in MVSN‐IR825 + MAUS group suggested the maximum alleviation of the hypoxic state.

### 2.5 In Vitro Anticancer Study

**Figure 6:**
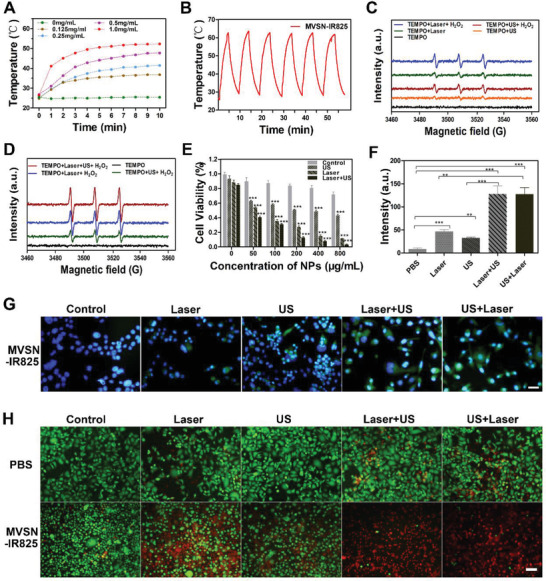
Anticancer therapeutic effect of MVSN‐IR825 in vitro (PC‐3 cells). A) Photothermal curve of MVSN‐IR825 in different concentrations under continuous laser irradiation (2 W cm^−2^). B) Photothermal stability curve of MVSN‐IR825 (5 mg mL^−1^) aqueous dispersion under laser irradiation (2 W cm^−2^). C) ESR spectrum of singlet oxygen in MVSN‐IR825 (100 µg mL^−1^) after laser irradiation (2 W cm^−2^, 1 min) and ultrasound irradiation (2 W, 1 min) mixed with and without H_2_O_2_. D) ESR spectrum of singlet oxygen in MVSN‐IR825 (100 µg mL^−1^) mixed with H_2_O_2_ after irritation with laser (2 W cm^−2^, 5 min), ultrasound (2 W, 5 min) and combination of the two. E) Cell viability of PC‐3 cells incubating with different concentrations of MVSN‐IR825 irradiated by laser and ultrasound (*n* = 5 per group, mean ± SEM, two‐way ANOVA, Bonferroni post‐test. In comparison to each control group, ***P < 0.001). F) Quantitative analysis of the fluorescence intensity of DCFH‐DA in each group (*n* = 3 per group, mean ± SEM, one‐way ANOVA, Tukey post‐test. **P < 0.01, ***P < 0.001). G) DCFH‐DA to detect the ROS produced by cells incubated with MVSN‐IR825 in each group (scale: 50 µm). H) Calcein‐AM/PI staining of PC‐3 cells incubated with MVSN‐IR825 after different treatments (scale: 100 µm). E–H) Laser irradiation at 2 W cm^−2^ for 5 min, and ultrasound irradiation at 2 W for 1 min. “Laser+ US” and “US + Laser” represented different order of using the two methods).

To explore the anticancer capability, the photothermal properties of MVSN‐IR825 were first assessed in vitro by using 825 nm laser irradiation. As shown in **Figure** **6**A, MVSN‐IR825 (1 mg mL^−1^) could be rapidly heated from ≈25 °C to ≈50 °C after 10 min laser irradiation with excellent photothermal stability (Figure 6B). The photothermal conversion of MVSN‐IR825 was calculated to be ≈32.1% (Figure S16, Supporting Information). To verify the photodynamic/sonodynamic capability of MVSN‐IR825, electron spin resonance (ESR) was employed to analysis the generation of ^1^O_2_ by laser or ultrasound irradiation. The result proved successful production of ^1^O_2_ of MVSN‐IR825 after laser and ultrasound (US) irradiation, and the effect could be further more intensified by adding H_2_O_2_ demonstrating oxygen‐assisted PDT and SDT (Figure 6C). The production of singlet oxygen increased with laser/ultrasound power and irradiation time (Figure S17, Supporting Information). Then, we further proved that combined laser and ultrasound irradiation did increase the production of ^1^O_2_, which indicated the feasibility of combined anticancer therapy (Figure 6D).

To further verify the anticancer effect at the cell level, a CCK‐8 assay was first carried out, which showed MVSN‐IR825 had no significant toxicity to PC‐3 cells even at high concentration (400 µg mL^−1^). Yet, under laser irradiation and ultrasound irradiation, the cell viabilities obviously decreased with elevating MVSN‐IR825 concentration (Figure 6E). The phenomenon could also be observed for PC‐3 cells irradiated by laser/ultrasound with increasing power and time (Figure S18A,B, Supporting Information). A concentration of 100 µg mL^−1^ MVSN‐IR825 with an obvious therapeutic effect but no obvious cytotoxicity at the same time was chosen for the following experiment. Similar to this, we chose “2 W cm^−2^” as the laser power intensity and “2 W” as the ultrasound power to conduct the following experiment in vivo. Next, 2,7‐dichlorofluorescein diacetate (DCFH‐DA) probe was used to verify the level of ROS at the cellular level and the brightness of green fluorescence represented the cellular ROS level. The strong green fluorescence demonstrated the intracellular mechanism of MVSN‐IR825 as a PDT and SDT agent, and the combined use of laser and ultrasound irradiation significantly increased the intracellular ROS level with no significant difference in the order of using these two methods (Figure 6G). Semiquantitative analysis of green fluorescence intensity of ROS by ImageJ pixel counting plugin also confirmed the result (Figure 6F). In addition, calcein acetoxy methyl ester (calcein‐AM, live cell staining) and propidium iodide (PI, dead cell staining) also verified the cellular therapeutic effect of MVSN‐IR825 (Figure 6H). Additionally, to further prove the role and contribution of PDT and PTT during laser irradiation, a CCK‐8 assay and calcein‐AM/PI staining revealed the lower cell viability of PTT than PDT, while the combined two contributed to the most efficient anticancer effect (Figures S19 and S20, Supporting Information).

To further evaluate the anticancer efficacy of our strategy in other cancer cells, 143B and AGS cells were also employed to examine the cellular ROS production and the cell viability by calcein‐AM/PI staining. A standard CCK‐8 assay suggested MVSN‐IR825 with a concentration of 100 µg mL^−1^ presented an obvious therapeutic effect but no obvious cytotoxicity was appropriate for the next experiments (Figure S21, Supporting Information). Then, DCFH‐DA staining in both 143B and AGS cells exhibited more green fluorescence in the laser + US and US + laser group than the laser and US group alone, which suggested the combined therapeutic method could produce more ROS at cell level (Figure S22, Supporting Information). Similarly, the calcein‐AM/PI staining reflected the most efficient anticancer results in the combined irradiation groups (Figure S23, Supporting Information).

### 2.6 Anticancer Efficacy and Safety Assessment In Vivo

**Figure 7:**
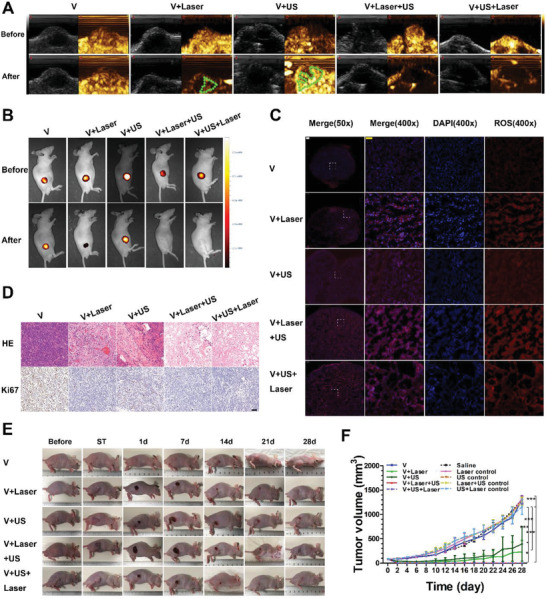
In vivo therapeutic effects of different methods in PC‐3 xenograft tumor‐bearing mice. A) Contrast‐enhanced ultrasound evaluation of tumors in each therapeutic group before and immediately after treatment. B) Bioluminescence monitoring tumor activity before and immediately after treatment. C) Dihydroethidium (DHE) staining of ROS in tumor tissues in each group. (The short white scale: 200 µm, the long yellow scale: 50 µm.) D) H&E and Ki67 staining of tumor in each experimental group (scale: 50 µm). E) Tumor morphology changes in mice at different time points after treatment. F) Tumor volume changes in mice at different time points (*n* = 3 per group, mean ± SEM, one‐way ANOVA, Tukey post‐test. *P < 0.05, ***P < 0.001).

With previous exciting results of enhanced nanoparticle accumulation and alleviated hypoxic state of tumor through marriage of using MVSN‐IR825 and MAUS delivery strategy, the anticancer efficacy in vivo was evaluated by using xenograft heterotopic model established by luciferase labeled human PC‐3 cell line. We wondered whether the combined laser and ultrasound irradiation of MVSN‐IR825 would inhibit tumors. According to the maximum accumulation of nanoparticles in tumors, anticancer therapy started 20 h after administration in all groups (saline group, laser control group, US control group, laser + US control group, US + laser control group, MVSN‐IR825 + MAUS group (V), V + laser group, V + US group, V + laser + US group, V + US + laser group. An infrared camera was used to monitor the temperature changes, which found that the local temperature of the tumor in V + laser group increased by ≈28 °C after laser irradiation, while that of the control group injected with normal saline increased by ≈6 °C (Figure S24, Supporting Information). Contrast‐enhanced ultrasound was used to evaluate the therapeutic effect immediately after treatment. An early rapid and high enhancement of contrast inside whole tumor before treatment indicated abundant microcirculation and active proliferation in the tumor, while low and heterogeneous residual contrast enhancement after treatment in V + laser group and V + US group indicated incomplete therapy of tumors, as showing in the green dotted area (**Figure** **7**A). However, there was no contrast enhancement in V + laser + US group and V + US + laser group, and the order of using the two methods did not affect the treatment result, which suggesting tumors were completely inactivated. In addition, as PC‐3 cells were labeled with luciferase in advance, the bioluminescence imaging could also be used to reflect the tumor activity. Similar to the results of contrast‐enhanced ultrasound, strong luminescence signal in tumor could be found in mice before treatment and in control group, which suggested the high activity of tumor cells. Yet, the significantly decreased luminescence signal in V + laser group and slightly decreased luminescence in V + US group both indicated tumors were partially destroyed, while V + laser + US group and V + US + laser group showed no bioluminescence signal inside tumor, which suggested the best therapeutic effect of tumor inactivation (Figure 7B). Next, ROS immunofluorescence revealed no red fluorescence expressed in each control group (Figures S25, Supporting Information), while obvious red fluorescence of ROS appeared in all treatment groups, and the ROS level in the two combined treatment groups were highest, which indicated the effectiveness of ROS production under laser and ultrasound irradiation based upon the intratumor accumulation of MVSN‐IR825 (Figures 7C). At the same time, H&E staining and Ki67 immunohistochemical staining of tumor tissues (Figure 7D; Figure S26, Supporting Information) and terminal deoxynucleotidyl transferase‐mediated dUTP‐biotin nick end labeling assay (TUNEL) and Ki67 immunofluorescence double staining were obtained (Figure S27, Supporting Information). Compared with each control group, the expression of Ki67 in the laser group and US group were relatively decreased, and the changes in the two combined treatment groups were much more obvious. The morphological (Figure 7E) and volume changes (Figure 7F) of tumors before treatment, immediately after treatment (statim, ST), 1 day, 7 days, 14 days, 21 days, and 28 days after treatment were recorded. The skin on the surface of the tumor turned white or slightly gray immediately after laser and US irradiation, and the tumor shrank obviously and formed scabs just 1 day after treatment. The scab area gradually decreased after 14 days, and returned to normal in ≈21 to 28 days. Specially, tumors in the V + laser + US group and V + US + laser group disappeared obviously on the first day after treatment, and there was no residue or recurrence in the observation period. In terms of safety evaluation, the weight of mice in each group was recorded, and there was no significant difference in weight among groups (Figure S23A, Supporting Information). Blood biochemical tests were performed in each treatment group (immediately after treatment group and 28 days after treatment group). It seems that the index of liver function, including the enzyme activity of alanine aminotransferase, aspartic transaminase, and alkaline phosphatase, increased immediately after treatment, especially in the two combined therapy groups (Figure S28B–F, Supporting Information). This sudden decrease of liver function might be due to stress response. All the important organs of mice in each group (immediately and 28 days after treatment) were subjected to H&E staining, which showed no obvious morphological damage (Figures S29 and S30, Supporting Information). In addition, the survival rate curve showed that laser or ultrasound therapy alone could prolong the survival rate but were not as effective as the combined therapy (Figure S31, Supporting Information).

Apart from the PC‐3 xenograft tumors, we further employed PC‐3 orthotopic model and the 143B xenograft model to evaluate the tumor inhibition effect of laser, US, and the combined irradiation. According to the previous exciting results, the anticancer therapy of the orthotopic model also started 20 h after intravenously administration, and the tumor bearing mice were randomly grouped as MVSN‐IR825 + MAUS group (V), V + laser group, V + US group, V + laser + US group, V + US + laser group. First, we used bioluminescence imaging to evaluate the tumor activity after different treatments. As depicted in Figure S32A of the Supporting Information, every nude mouse showed strong luminescence signal at lower abdomen before treatment indicating the high activity of the orthotopic tumor cells. Yet, the luminescence signal decreased immediately after laser and ultrasound irradiation, while the two combined therapy groups with no luminescence signal in the lower abdomen, which indicated the efficient tumor inhibition effect. Additionally, hematoxylin and eosin (H&E), Ki67, TUNEL, and ROS staining verified the efficient anticancer therapy basing on the successful accumulation of the nanoparticles (Figure S33B, Supporting Information). Similarly, the 143B xenograft model also demonstrated efficient tumor inhibition in bioluminescence imaging (Figure S36A, Supporting Information) and immunohistochemical and immunofluorescence staining (Figure S36B, Supporting Information). Then the size of the PC‐3 orthotopic tumors was monitored by clinical ultrasound until 14 days after treatments (Figure S33E, Supporting Information). Significant difference appeared between V group and two combined therapy groups, thus verifying the best result of tumor inhibition (Figure S33C, Supporting Information). Moreover, no significant difference on the body weight (Figure S33D, Supporting Information) and H&E staining of major organs was observed in each group (Figure S34, Supporting Information). The similarly efficient therapeutic efficiency could also be found in 143B xenograft model (Figure S36C–E, Supporting Information).

## 3 Conclusion

In this work, the delivery efficiency of nanoparticles in solid prostate tumors was remarkably enhanced by optimizing the structure of nanoparticles as virus‐mimic surface topology to enhance nano–bio interactions and utilizing microbubble‐assisted low‐frequency ultrasound as a passive targeting method to improve permeability of biological barriers. Through the novel combination of intrinsic surface topological superiority of nanoparticles and extrinsic ultrasonic stimulation, we have demonstrated dramatically enhanced intratumor penetration and distribution of MVSN‐IR825. Furthermore, our theranostic nanoplatform have showed improved triple‐modal imaging (PAI/FI/MRI) and trimodal anticancer therapeutic functions (PTT/PDT/SDT) basing upon the increased intratumor accumulation of nanoparticles. Overall, this strategy offers an innovate delivery paradigm that promises to solve the critical puzzle in clinical cancer nanomedicine, which paves a new way for developing efficient cancer theranostic modality. Though we have employed PC‐3/143B/AGS cells in vitro, PC‐3/143B xenograft model and PC‐3 orthotopic model in vivo, future efforts should explore the university of our strategy in patient‐derived xenograft models or other cancer types, which will be valuable in facilitating its future clinical translation.

## 4 Experimental Section

##### Materials

Hexadecyltrimethylammonium bromide (CTAB, 99%) was purchased from Sigma‐Aldrich (USA). Cyclohexane, sodium hydroxide (NaOH, 99%), ethanol (99.8%), ferric chloride (FeCl_3_∙6H_2_O, 99%), trisodium citrate (99%), NaAc (99%), ethylene glycol (99.8%), and ammonium hydroxide (28%) were obtained from Shanghai Chemical Co., Ltd. (Shanghai, China). Tetraethylorthosilicate (TEOS, 99%), (3‐Aminopropyl) triethoxysilane (APTES, 99%), triethanolamine (TEA, 98%), *N*‐(3‐dimethylaminopropyl)‐*N*′‐ethylcarbodiimide hydrochloride (EDC, 98%), and *N*‐fydroxysuccinimide (NHS, 98%) were purchased from Aladdin Industrial Co., Ltd. (Shanghai, China). All chemicals were used as received without further purification. IR825, FITC, and PEG‐COOH (MW 2000 Da) were purchased from Xinqiao Biotechnology Co., Ltd. (Hangzhou, China). Hydrogen peroxide (H_2_O_2_) was purchased from Saigo Reagent Co., Ltd. (Hangzhou, China). Calcein‐AM and PI were purchased from Shanghai Maokang Biotechnology Co., Ltd. (Shanghai, China). CCK‐8 was purchased by Dojindo Laboratories (Kyushu, Japan). DAPI and DCFH‐DA were purchased from Beyotime Biotechnology Co., Ltd. (USA). TUNEL, Ki67, H&E, HIF‐1*α* antibody, and ROS detection agent DHE were purchased from Wuhan Seville Biotechnology Co., Ltd. (Wuhan, China). Ham's F‐12K nutrition medium (Kaighn improved) containing l‐glutamine medium (F‐12K) and phosphate buffered saline (PBS) were purchased from Shanghai Yuanpei Biotechnology Co., Ltd. (Shanghai, China). Fetal bovine serum (FBS), penicillin/treptomycin and trypsin were provided by Gibco Life Technologies Co., Ltd. (Great Island, USA). Mycoplasma prevention was purchased from Invitrogen Co. (USA). PC‐3, AGS, and 143B cells were purchased from the cell bank of the Shanghai Institute of Biochemistry and Cell Biology, Chinese Academy of Sciences. Luciferase labeled PC‐3 cells and 143B cells were constructed by Hanheng Biological Company (Shanghai, China).

##### Synthesis of Fe_3_O_4_ Nanoparticles

For the synthesis of Fe_3_O_4_,^[^ ^49^ ^]^ FeCl_3_∙6H_2_O (1.62 g), trisodium citrate (0.65 g), and NaAc (3.0 g) were dissolved in ethylene glycol (50 mL) with fully stirring. Then, the obtained solution was transferred and sealed into a Teflon‐lined autoclave (100 mL), which was heated at 180 °C for 12 h. The products were washed with deionized water and ethanol for several times, and then dried under vacuum at 40 °C for 24 h.

##### Synthesis of the MSSNs

For the synthesis of MSSNs, a modified Stöber method was applied. Generally, Fe_3_O_4_ (40 mg) was dispersed in a solution containing ethanol (80 mL), deionized water (10 mL), and aqueous ammonia (1.8 mL), with stirring for 1 h. Then, TEOS (2 mL) was added to the above system with stirring for another 8 h. The products were collected by centrifugation and washed with water and ethanol several times. The obtained products were dried in vacuum at 40 °C for 24 h.

##### Synthesis of the MMSNs

The MMSNs were synthesized by a previously reported procedure. Briefly, Fe_3_O_4_ (50 mg) was dispersed in water (60 mL) by sonication. Afterward, CTAB (6.0 g) and TEA (1.2 mL, 25 wt%) were added to the above solution and stirred slowly (≈ 300 rpm) at 60 °C for 2 h. Then, mixture (20 mL) of TEOS (4 mL) in cyclohexane (16 mL) was added to the solution and kept at 60 °C in an oil bath with slow stirring (≈300 rpm) for 12 h. The obtained products were then collected by centrifugation and washed with water and ethanol for several times. The obtained MMSNs were dried in vacuum at 40 °C for 24 h for further use.

##### Synthesis of the MVSNs

MVSNs were synthesized by the single‐micelle epitaxial growth method in a biphase system as previously reported. ^[^ ^31^ ^]^ Typically, Fe_3_O_4_ (60 mg) was dispersed in water (50 mL) by sonication. Afterward, CTAB (1.5 g) and 0.8 mL of NaOH (0.1 m) were added to the solution and stirred slowly (≈300 rpm) at 60 °C for 2 h. Then, the mixture (20 mL) of TEOS (4 mL) in cyclohexane (16 mL) was added to the above solution and kept at 60 °C with slow stirring (≈300 rpm) for 48 h. The obtained samples were collected by centrifugation and washed with water and ethanol for several times. The obtained MVSNs were dried in vacuum at 40 °C for 24 h for further use.

##### Synthesis of Amino Group‐Modified MSSN, MMSN, and MVSN

The amino groups were grafted on the three kinds of nanoparticles by post‐synthetic modification method. First, the nanoparticles (100 mg) was dispersed in ethanol (40 mL) by ultrasonic. Then, 3‐aminopropyltriethoxysilane (200 µL) was added, and the solution was refluxed for 24 h under the nitrogen atmosphere with continuous stirring. The products were collected by centrifuging and washed with ethanol, and water, and dried in vacuum at 40 °C for 24 h.

##### Synthesis of PEG‐Modified MSSN, MMSN, and MVSN

For the synthesis of PEG‐modified nanoparticles, PEG‐COOH (25 mg) was dissolved in water (10 mL) containing 8 mg of EDC, 5 mg of NHS and stirred for 2 h. The prepared amino group‐modified nanoparticles (20 mg) predispersed in 10 mL of water was added into the activated PEG‐COOH solution, followed by another 24 h reaction. The products were collected by centrifugation and washed with water. The PEG‐COOH was linked to the surface of nanoparticles mainly by the formation of amido covalent bonds.

##### Synthesis of FITC‐Modified MSSN, MMSN, and MVSN

For the synthesis of FITC‐labeled nanoparticles, PEG‐modified MVSNs (MSSNs or MMSNs) (20 mg) was dispersed in ethanol (10 mL), and 1 mL of FITC ethanol solution (0.5 mg mL^−1^) was added. The reaction mixture was stirred for 12 h in the dark. The products were then separated by centrifugation and washed with ethanol. After drying in vacuum at 45 °C, the FITC‐labeled products were obtained. The FITC was linked to the surface of nanoparticles mainly by the formation of thiourea derivative.

##### Synthesis of IR825‐Modified MSSN, MMSN, and MVSN

For the synthesis of MVSN‐IR825 (MSSN‐IR825/MMSN‐IR825), PEG‐modified MVSNs (MSSNs, MMSNs) (20 mg) were dispersed in ethanol (20 mL). Then, 1.0 mL of IR825 ethanol solution (0.5 mg mL^−1^) was added to the mixture and stirred for 12 h in the dark. The products were then separated by centrifugation and washed with ethanol. After drying in vacuum at 45 °C, MVSN‐IR825 (MSSN‐IR825, MMSN‐IR825) were obtained. The IR825 was linked to the surface of nanoparticles mainly by physical interactions.

##### Measurements and Characterization

SEM imaging was performed with a Hitachi S4800 7593‐H. TEM imaging was carried out with an FEI Tecnai G2 F20. HAADF‐STEM imaging and EDX elemental mapping were characterized by a JEOL 2010F microscope operating at 200 kV. Magnetic hysteresis loops were performed by using vibrating sample magnetometer (LakeShore7404). The contents of singlet oxygen (^1^O_2_) were determined by an ESR spectrometer (Bruker A300). The UV–vis–NIR absorption spectra were determined by a BioTek Epoch microplate spectrophotometer (Vermont, USA). The absolute content of iron was determined by inductively coupled plasma mass spectrometry (ICP‐MS) (Agilent 7800, USA). Particle size distribution data were measured by a dynamic light scattering particle size analyzer (Brookhaven, USA). Thermogravimetric (TG) analysis was carried out from room temperature to 800 °C under nitrogen flow using a thermal analyzer (TG/DTA7300, Seiko Instruments Inc., Chiba, Japan). Fourier transform infrared (FTIR) spectrum was measured by an FTIR spectrometer (Thermo Scientific Nicolet iS5, Waltham, MA). Flow cytometry was carried out using a CytoFLEX Flow cytometer (Beckman Coulter, Inc. USA). The low‐frequency ultrasonic generator was manufactured by Jiangsu Baihang Ultrasonic Equipment Co., Ltd. (Wuxi, China). Fluorescence microscopy images were obtained with an Olympus IX 70 (Japan). In vivo FI was administered with a Visque In vivo Elite optical in vivo imaging system (Vieworks, Inc. South Korea). PAI was completed by Visualsonics (Vevo 2100, Canada). MRI examination was performed using a 7.0 T animal MRI instrument (Chenguang Medical Technologies Co., Ltd., Shanghai, China). The real‐time thermal imaging was recorded by an infrared camera (AnalyzIR, Shanghai, China). The ultrasonic parameters were as follows: the diameter of the transducer was about 1.25 cm, and the irradiation area was ≈1.2 cm^2^; the acoustic power of the transducer can be adjusted to 1, 1.5, and 2 W, which has been previously determined by using radiation force balances (RFB, IEC 61161: 2013). Thus, the corresponding acoustic intensity was 0.83, 1.25, and 1.67 W cm^−2^, while the acoustic power was 0.160, 0.196, and 0.227 MPa, respectively. The transducer frequency was 500 kHz, the impulse wave was the square wave, the pulse‐repetition frequency was 0.001 kHz, the duty cycle was 50%, the pulse width was 500 msec. The NIR laser with a wavelength of 825 nm was manufactured by Changchun New Industries Optoelectronics Technology Co., Ltd. (Changchun, China). The spot area can be adjusted between 0.5 and 2 cm^2^, and the power density can be adjusted according to the spot area. Contrast‐enhanced ultrasound was performed via a clinical abdominal probe of Mylab twice with transducer frequency of 8 MHz (Esaote, Italy). A GE Logic E9 ultrasound system (GE Healthcare, Milwaukee, WI, USA) with an 18.0 MHz frequency transducer (GE Healthcare) was used to monitor the growth of the orthotopic prostate cancer.

##### Conjugation Rate Detection

The conjugation rate (CR%) and the drug loading (DL%) were calculated as following details. The absorption spectra of IR825 with different concentrations in ethanol solution were detected and the linear equation between the concentration and the absorbance was developed. Next, the supernatant of MSSN‐IR825 (MMSN‐IR825/MVSN‐IR825) in synthesis procedure were collected to measure the absorbance, and the absorbance of those were 2.481/2.237/0.734, respectively. Then, the concentration of IR825 in the supernatant was calculated by substituting the absorbance into the standard curve equation, and the supernatant concentration of MSSN‐IR825/MMSN‐IR825/MVSN‐IR825 was 0.0588/0.0538/0.023 mg mL^−1^, respectively. Thus, the volume of the supernatant (≈32.5 mL) was multiplied by the concentration to obtain the mass of IR825 in the supernatant.

The CR% was calculated according to the formula

(1) $$\mathrm{CR}\%=\frac{W-W_{s}}{W}\times 100\%$$

The DL% was calculated according to the formula

(2) $$DL\%=\frac{W-W_{s}}{W_{a}}\times 100\%$$

The *W* represented the mass of IR825, which was about 6 mg, *W*_a_ represented the total mass of MSSN‐IR825 (MMSN‐IR825/MVSN‐IR825), and *W*_s_ represented the mass of IR825 in the supernatant.

##### Cell Culture

PC‐3, AGS, and 143B cells were cultured in F‐12K medium (F‐12K, Chinese source) supplemented with 10% heat‐inactivated FBS, streptomycin (100 U mL^−1^), and penicillin (100 U mL^−1^), and mycoplasma prevention (1:500) and cultured in a 37 °C, 5% CO_2_‐humidified incubator.

##### Toxicity of Nanoparticles

The standard CCK‐8 scheme was performed to evaluate the cytotoxicity of nanoparticles. PC‐3, AGS, and 143B cells were inoculated in a 24‐well plate at a density of 1 × 10^5^ cells per well and cultured for 24 h at normal conditions (37 °C humidified 5% CO_2_ atmosphere). According to the experimental conditions, the groups were divided according to different concentrations of nanoparticles. After 24 h of closed incubation, the culture medium of each group was discarded and replaced with 400 µL of fresh F‐12K medium without fetal bovine serum containing 10% CCK‐8 for another 4 h incubation. Next, the supernatant CCK‐8 medium was transferred to a new plate and the optical absorbance density per well was detected at 450 nm by the microplate reader and compared with that of the control group, and the survival rate was calculated according to the following formula

(3) $$\mathrm{Cell}\;\mathrm{viability}\left(\%\right)=\left[\frac{\mathrm{As}-\mathrm{Ab}}{\mathrm{Ac}-\mathrm{Ab}}\right]\times 100\%$$

In the formula, “As, Ab, and Ac” represented the absorbance of experimental well with nanoparticles and cells, the blank well without nanoparticles and cells, and the control well with cells but without nanoparticles, respectively.

For in vivo biosafety of nanoparticles, major organs were harvested 24 h after intravenously injection of three nanoparticles and H&E staining was employed to evaluate the tissue damage.

##### Cellular Endocytosis Study

PC‐3 cells were inoculated into 24‐well plates at a density of 5 × 10^5^ cells per well and cultured for 24 h. Next, FITC‐labeled MSSN‐IR825, MMSN‐IR825, and MVSN‐IR825 were added at a concentration of 100 µg mL^−1^ and incubated for 30 min, 1 h, 3 h, 6 h, and 12 h. To explore the effect of ultrasound stimulation on cell uptake, the three nanoparticles mixed with clinical ultrasound microbubbles or PBS were added into PC‐3 cells in ultrasound stimulation groups or control groups, respectively. Details of ultrasound irradiating cells in 24‐well plate were presented in Figure S38 of the Supporting Information. Then cells were irradiated by the low‐frequency ultrasound and then incubated for another 6 h. Following the incubation period, the culture medium was removed and cells were softly washed with cold PBS more than three times to remove free nanoparticles. The nuclei were stained with DAPI to observe their relationship with green fluorescent FITC‐labeled nanoparticles via fluorescence microscopy. The average fluorescence intensity of FITC was analyzed by ImageJ pixel counting plugin.^[^ ^50^ ^]^ In addition, the quantitative data of FITC reflecting cell phagocytosis were obtained by flow cytometry. Similarly, 143B and AGS cells were also used to evaluate the cellular endocytosis for 6 h incubation with FITC‐labeled MSSN‐IR825, MMSN‐IR825, MVSN‐IR825, and the effect of microbubble‐assisted ultrasound following the same method.

##### Animal Model

BALB/c nude mice were purchased from the Experimental Animal Center of the Sixth People's Hospital of Shanghai. The animal experiments were carried out according to the scheme approved by the Animal Protection and Utilization Committee of the Sixth People's Hospital affiliated with Shanghai Jiao Tong University (Animal Welfare Ethics (Acceptance No: DWLL2019‐0403)). For the xenograft heterotopic model of prostate cancer, male nude BALB/c mice aged 4 weeks were selected, and luciferase labeled 5 × 10^6^ PC‐3 cells in 100 µL PBS were subcutaneously injected into the right lower limb joints of each mouse to establish the subcutaneous xenografts. The size of the tumor was recorded with a digital caliper until 28 days after treatment (*n* = 3). The tumor volumes were calculated as: *V* = 0.5 × (length) × (width)^2^. When the tumor grew to a uniform size of about 80 mm^3^, the date was designated as 0 day, which indicates the therapeutic process began.

For the orthotopic tumor model, 5‐week male nude BALB/c mice were selected. The surgical orthotopic implantation method was used to establish the orthotopic primary prostate tumor models as previous reported.^[^ ^51^ ^]^ The previous subcutaneous xenograft tumors with luciferase labeled PC‐3 cells were used as donor tumors. A mass with a volume of about 1 mm^3^ was buried into the capsule of a sterile environment. The growth of the tumor in situ was dynamically monitored by using clinical ultrasound with a probe of 18 MHz frequency. In addition, ultrasound‐guided cross location method was used to display the surface projection of the orthotopic primary tumor, where low‐frequency ultrasound irradiated in the meantime of intravenous injection to promote the enrichment of nanoparticles in orthotopic tumor. Intravenous administration started when the diameter of tumor reached ≈4–5 mm. The size of tumor in situ was measured by clinical ultrasound twice a day until 14 days after treatment (*n* = 3).

For the xenograft model of osteosarcoma, luciferase labeled 5 × 10^6^ 143B cells in 100 µL PBS were subcutaneously injected into the right lower limb joints of 4‐week male nude BALB/c mice to establish the subcutaneous xenografts. The tumor size was recorded with a digital caliper until 7 days after treatment (*n* = 3). The tumor volumes were calculated as: *V* = 0.5 × (length) × (width)^2^. When the tumor grew to ≈60–80 mm^3^, the date was designated as 0 day indicating the experiment started.

##### In Vivo Blood Circulation Experiment

The MSSN‐IR825/MMSN‐IR825/MVSN‐IR825 in saline were injected into nude mice (30 mg kg^−1^) through the tail vein. Blood samples were periodically collected from the tail vein specified time points (2 min, 1 h, 2 h, 3 h, 4 h, 8 h, 16 h, and 24 h) in eppendorf tubes after anticoagulant treatment and stored at −20 °C before ICP‐OES analysis of Fe element. According to the fist‐order elimination kinetics, the curve could be transformed to a linear plot by using the natural logarithm of the concentration as the vertical coordinate. Blood circulation half life could be obtained by using the formulas:

(4) $$\log C=\log C_{0}-\frac{k}{2.303}t$$

(5) $$t_{1/2}=0.693/k$$

##### Microbubble‐Assisted Low‐Frequency Ultrasound Delivery Strategy

The clinical contrast agent SonoVue with a mass of 59 mg containing sulfur hexafluoride gas and powder per vial can be solved with 5 mL saline. When made up into a solution, the gas can be trapped in tiny bubbles (a mean diameter of about 2.5 µm) called “microbubbles” in suspension in a liquid. 50 µL microbubble suspension was mixed together with nanoparticles (60 mg kg^−1^) to intravenously inject into tumor‐bearing mice via tail vein. It was a remarkable fact that the low‐frequency ultrasound irradiation of the tumor site was administered at almost the same time as intravenous injection. Specially, the speed of intravenous injection was as slow as controlling the duration for ≈1 min and the ultrasound irradiation time was about 2 min. For the alignment with the subcutaneous tumor, the mice were placed through a circular plastic ring upon an iron stand. In particular, a 1.5 mL eppendorf tube was cut as a prop to place between the ultrasound probe and the tumor. The surface of the ultrasound probe and the modified tube inside was filled with gel. This special tube would align the subcutaneous tumor so that the tumor could be fully immersed in the gel meanwhile not too much surrounding normal tissue was irradiated. Details of alignment with the subcutaneous tumor were presented in Figure S40 of the Supporting Information.

##### Multiple Imaging In Vivo

Visual Sonic Vevo‐2100 LAZR system designed for small animal was employed in PAI. For in vitro PAI of nanoparticles, the above nanoparticles at different concentrations were injected into polyethylene capillaries immersed in coupling gel (centrifugation at 1500 rpm for 10 min to remove air). For in vivo PAI of tumor site, thick coupling gel after removing air was covered on subcutaneous tumor. The quantified 3D PA signals were measured in the region of interest tumor area and the oxygen saturation of tumors was measured in the “Oxyhemo” mode. It was worth noting that the PA gain, time gain compensation should be consistent each mouse. Visque in vivo Elite optical imaging system was used in vivo FI, and the ICG imaging channel was selected to detect the fluorescence intensity of MVNP‐IR825 accumulated in tumor. For bioluminescence imaging to detect the luciferase labeled PC‐3 cell activity, 150 mg mL^−1^ of fluorescein substrate potassium salt was previously injected intraperitoneally before anesthesia. Then 15 min later, the luminescence imaging channel was selected to measure the luminescence signal of tumor in units of photons/s/cm^2^/steradian before or immediately after treatment. MRI was performed using T2 sequence with a 7.0 T magnetic resonance scanner for special small animal use. Tumor‐bearing mice were scanned with a special coil set for small animal imaging before and 20 h after intravenous injection, and the same slice of each mouse should be selected for comparison. For in vivo infrared photothermal imaging, an AnalyzeIR thermal imager was used to monitor the real‐time temperature change in tumors irradiated by an 825 nm near‐infrared laser.

##### Biodistribution Study

Tumor‐bearing mice were randomly divided to the following experimental groups: MSSN‐IR825 group, MSSN‐IR825+ MAUS group, MMSN‐IR825 group, MMSN‐IR825+ MAUS group, MVSN‐IR825 group, MVSN‐IR825+ MAUS group. Here “MAUS” was shorted for microbubble‐assisted low‐frequency ultrasound irradiation. The Prussian blue staining was used to detect the iron distribution and penetration in tumors. The quantitative data of delivery efficiency were represented as a percentage of the injected dose (%ID) in each tissue at designed time point, respectively. In detail, major organs and tumors were intactly harvested, rinsed by saline and then weighted, and next solubilized in sodium hydroxide (2 m) for 24 h and then in aqua regia under heating for 2 h. Each sample was diluted with DI water and diluted to certain volumes to prepare for ICP‐MS analysis. The baseline Fe content in each organ of untreated mice were also measured and subtracted. Three mice were used for each group and each sample was measured with two repeats.

##### Evaluation of Oxygen Production Capacity

For in vitro evaluation of oxygen generation, a dissolved oxygen meter was employed to detect the changes of oxygen solubility. The MVSN‐IR825 was diluted with degassed water. The oxygen solubility detector was turned on and calibrated in air. Then, the probe was placed in the liquid middle layer, and the top layer was sealed with 5 mL of paraffin oil to prevent external oxygen from dissolving into the water, which would affect the accuracy of the experimental data. After the data were stable, hydrogen peroxide was added, and the changes of oxygen solubility were recorded for 30 min. The resuspension concentration of MVSN‐IR825 in degassed water was 1 mg mL^−1^, and the concentration of H_2_O_2_ was 20 µmol L^−1^. For in vivo evaluation of oxygen production in tumor, PAI was employed to detect the oxyhemoglobin saturation of tumor, and HIF‐1*α* staining was administered to show the oxygen level inside tumor.

##### Photothermal Conversion

To investigate the photothermal conversion efficiency of MVSN‐IR825, 100 µL of MVSN‐IR825 (5 mg mL^−1^) in a 96‐well plate was irradiated with an 825 nm near‐infrared laser (2 W cm^−2^). After 5 min of irradiation, the laser was turned off, and the temperature of the solution was recorded over time. The inverse formula of the natural logarithm of the cooling time and the driving temperature was linearly fitted (3). According to the literature, the photothermal conversion efficiency (*η*) of MVSN‐IR825 was calculated by using the following formulas^[^ ^52^ ^]^

(6) $$\eta =\frac{hA\Delta T_{\max}-Q_{S}}{I\left(1-10^{-A_{\lambda }}\right)}$$

(7) $$\tau_{s}=\frac{m_{D}C_{D}}{hA}$$

(8) $$t=-\tau_{s}\ln\left(\theta \right)$$

(9) $$\theta =\frac{\Delta T}{\Delta T_{\max}}$$

(10) $$Q_{s}=hA\Delta T_{H_{2}O}$$

In formula (6), Δ*T*_max_, representing the maximum temperature change of MVSN‐IR825 was 37.1 °C (see Figure S16B, Supporting Information). In formula (10), $\Delta T_{H_{2}O}$, showing the maximum temperature change of water, was 0.9 °C (see Figure S16B, Supporting Information). *Q*_S_ was the heat of light absorbed by the solvent (water) per second. I, representing laser power, was 0.4 W (the area irradiated by the laser per hole in a 96‐hole plate was ≈0.2 cm^2^), and *A*_*λ*_, the absorbance of MVSN‐IR825 at 825 nm, was 1.882. In formula (2), the mass of water was 0.1 g, the heat capacity of water is 4.2 J g^−1^ K^−1^, and *τ*_s_ is the heat transfer time constant of 120 s (see Figure S16D, Supporting Information). The *hA* calculated according to formula (7) was 0.35 × 10^−2^ W K^−1^. According to the obtained data and formula (6), the photothermal conversion efficiency (*η*) of MVSN‐IR825 was calculated to be 32.1%.

##### Quantitative Detection of ROS in vitro

For ESR analysis of ROS, ^1^O_2_ generation was detected by 2, 2, 6, 6‐tetramethylpiperidine nitrogen‐oxide (TEMPO). The characteristic peak signal was detected by ESR. In order to evaluate the effect of combined treatment on ^1^O_2_ generation, 200 µL MVSN‐IR825 (100 µg mL^−1^) was mixed with 200 µL H_2_O_2_ (1 × 10^−3^ m) and 100 µL TEMPO, and then exposed to laser irradiation (825 nm, 2 W cm^−2^, 1 min), US irradiation (500 kHz, 2 W, 1 min), and the above two combined treatment. In order to investigate the oxygen‐assisted PDT and SDT, H_2_O_2_ was removed to make a comparison. To further explore the effect of laser/ultrasound irradiation power intensity and time on ^1^O_2_ generation, ESR analysis was administered under the designed irradiation condition.

##### In Vitro Anticancer Therapy

PC‐3 cells, 143B, and AGS cells were seeded in 24‐well plates and incubated with MVSN‐IR825 (100 µg mL^−1^) for 24 h, and then treated with laser and ultrasound irradiation according to groups (PBS, Laser, US, Laser+ US, US+ Laser). In detail, low‐frequency ultrasound irradiation was administered upward from the bottom of the plate, and thick coupling gel was filled between the transducer and the well bottom (Figure S38, Supporting Information). The parameters of low‐frequency ultrasound irradiation were 2 W for acoustic power, and 1 min for irradiation time. Laser irradiation was administered downward from the top of the plate, during which the spot size of laser was regulated to cover the cells per well and the laser power could be adjusted according to the irradiation area to maintain a certain laser power density. The parameters of 825 nm NIR laser irradiation were 2 W cm^−2^ for power intensity, and 5 min for irradiation time. In order to explore the contribution of PDT and PTT in laser irradiation, an ice box previously stored at −20 °C was placed under the 24‐well plate to maintain a constant temperature for PDT procedure during laser irradiation; and vitamin C (10 × 10^−3^ m) were added together with nanoparticles into PC‐3 cells for 24 h incubation for PTT procedure according to the previous report.^[^ ^53^ ^]^ The anticancer effect in vitro was administered by CCK‐8 test for cell viability evaluation, the calcein‐AM (4 × 10^−6^ m) and propidium iodide PI (4.5 × 10^−6^ m) staining for live/dead cell fluorescence imaging, and the DCFH‐DA (10 × 10^−6^ m) staining and DAPI (2 × 10^−3^ m) staining for cellular ROS fluorescence imaging.

##### In Vivo Anticancer Therapy

Subcutaneous xenograft prostate cancer model was constructed by using luciferase‐labeled human PC‐3 cell line in nude BALB/c mice. Anticancer therapy was performed when the tumor volume reached 80 mm^3^. The tumor‐bearing mice were randomly divided into ten groups: saline group (saline injection intravenously without any treatment), laser control group (intravenous saline injection and laser irradiation), US control group (intravenous saline injection and US irradiation), laser+ US control group (intravenous saline injection and laser irradiation, then US irradiation), US+ laser control group (intravenous saline injection and US irradiation, then laser irradiation), V group (administration with MVSN‐IR825 and microbubble‐assisted ultrasound delivery strategy but without any treatment, and abbreviated to V group), V+ laser group (administration of MVSN‐IR825 and microbubble‐assisted ultrasound delivery strategy and laser irradiation), V+ US group (administration of MVSN‐IR825 and microbubble‐assisted ultrasound delivery strategy and US irradiation), V+ laser+ US group (administration of MVSN‐IR825 and microbubble‐assisted ultrasound delivery strategy, first laser irradiation, and then ultrasound irradiation), V+ US+ laser group (administration of MVSN‐IR825 and microbubble‐assisted ultrasound delivery strategy, first ultrasound irradiation, and then laser irradiation). Laser irradiation (825 nm, 2 W cm^−2^, and 10 min) should regulate the spot size to cover the tumor area and the laser power could be adjusted according to the irradiation area to maintain the laser power density. As for low‐frequency ultrasound irradiation (2 W, 10 min), the detailed procedure of alignment with the subcutaneous tumor was listed according to Figure S40 of the Supporting Information. The body weight and tumor volume of mice were observed and monitored. Blood samples was obtained to analyze the biochemical indexes of liver and kidney function immediately after treatment (*n* = 3) and 28 days after treatment (*n* = 3), and then the mice in each group were euthanized by cervical dislocation after the injection of excessive anesthetic. The main organs (heart, liver, spleen, lung, and kidney) were harvested for H&E staining to determine the safety of the treatment. For H&E, TUNEL, Ki67, and DHE staining, tumor tissues were dissected sixth hours after treatment. The rest of the mice in each group (*n* = 5) were observed to 60 days, and a survival rate curve was constructed to assess the therapeutic effect. For PC‐3 orthotopic tumor‐bearing mice, the laser irradiation and ultrasound irradiation were conducted in a sterile environment and the details were presented in Figure S41 of the Supporting Information. The body weight and tumor volume of mice were observed and monitored until 14 days after treatment, and the major organs 14 days after treatment were harvested for H&E staining to determine the safety of the engineered nanoparticles.

For 143B subcutaneous xenograft tumor‐bearing mice, the detailed procedure of alignment with the subcutaneous tumor was the same as the PC‐3 xenograft model previously depicted in Figure S39 of the Supporting Information. The body weight and tumor volume of mice were observed and monitored until 7 days after treatment, and the main organs 7 days after treatment were harvested for H&E staining to determine the safety of the treatment.

##### Statistical Analysis

All experiments were performed in at least triplicate and data were expressed as the mean ± standard error of the mean (SEM). The difference between two groups was analyzed by independent sample *t*‐test. The differences among multiple groups were analyzed by one‐way ANOVA followed by Tukey post‐test, and two‐way ANOVA followed by Bonferroni post‐test. The log‐rank (Mantel–Cox) test was used to compare the survival curves. GraphPad Prism software version 5.01(GraphPad Software Inc., CA, USA) was used for all statistical analyses. The statistical significance was indicated as *P* < 0.05, and expressed as *P < 0.05, **P < 0.01, ***P < 0.001.

## Conflict of Interest

The authors declare no conflict of interest.

## Supporting information

Supporting Information

## Data Availability

Research data are not shared.
