# Supplementary material for: Marriage of Virus‐Mimic Surface Topology and Microbubble‐Assisted Ultrasound for Enhanced Intratumor Accumulation and Improved Cancer Theranostics
Source: Adv Sci (Weinh). 2021 May 14;8(13):2004670. doi: 10.1002/advs.202004670 (PMC8261514; doi:10.1002/advs.202004670)
Supplement: Supplementary file 1 — Supporting Information [file ADVS-8-2004670-s001.pdf]

© 2021 Wiley-VCH GmbH

ADVANCED SCIENCE
Open Access

Supporting Information

for *Adv. Sci.,* DOI: 10.1002/advs.202004670

Marriage of Virus-mimic Surface Topology and Microbubble-assisted Ultrasound for Enhanced Intratumor Accumulation and Improved Cancer Theranostic

Zheying Meng, Yang Zhang, E Shen, Wei Li, Yanjie Wang, Krishnan Sathiyamoorthy, Wei Gao, Michael C. Kolios, Wenkun Bai, Bing Hu*, Wenxing Wang*, and Yuanyi Zheng**

Supporting Information

Marriage of Virus-mimic Surface Topology and Microbubble-assisted Ultrasound for Enhanced Intratumor Accumulation and Improved Cancer Theranostic

Zheying Meng, Yang Zhang, E Shen, Wei Li, Yanjie Wang, Krishnan Sathiyamoorthy, Wei Gao, Michael C. Kolios, Wenkun Bai, Bing Hu*, Wenxing Wang*, Yuanyi Zheng**

Supplementary Figures

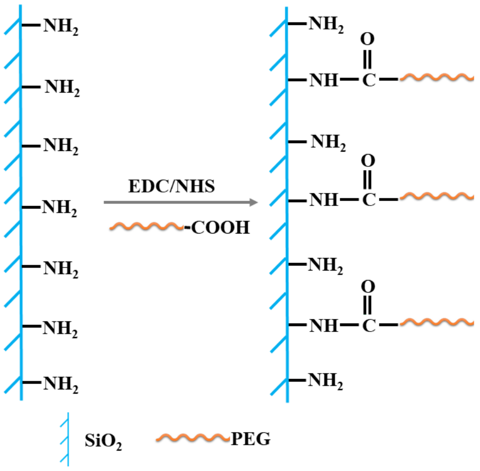

Figure S1. Schematic illustration of the surface modification with PEG-COOH.

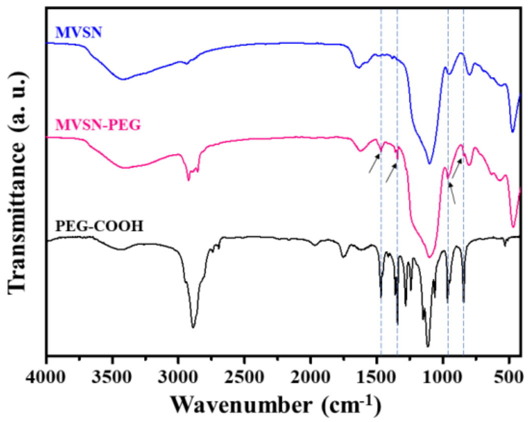

Figure S2. FTIR spectra of the PEG-COOH, MVSN and MVSN-PEG. The characteristic frequency at 1466, 1346, 960, 841 cm^{-1} can be assigned to CH_2 vibration of PEG, indicating that the surface of the MVSN was successfully modified with PEG.

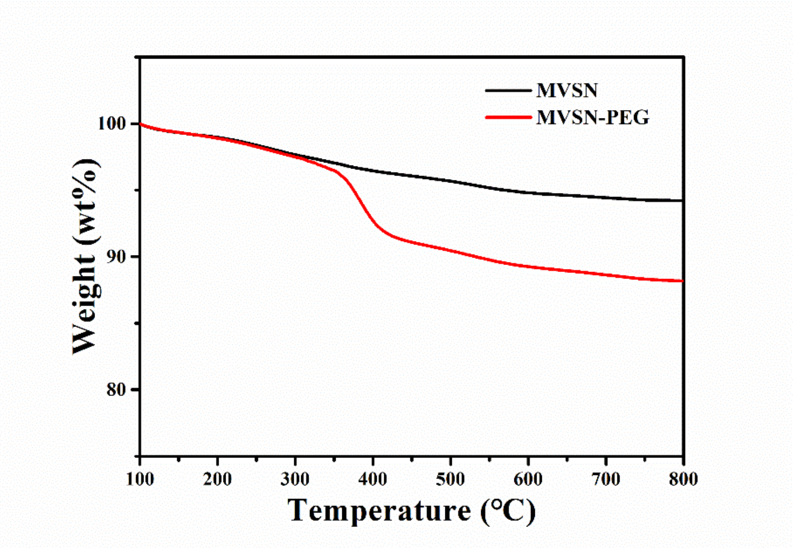

Figure S3. Thermogravimetric analysis of MVSN and MVSN-PEG. The weight loss of MVSN was calculated to be 5.0%, while it increased to 11.06% for MVSN-PEG, which further confirmed successful surface modification of PEG in MVSNs.

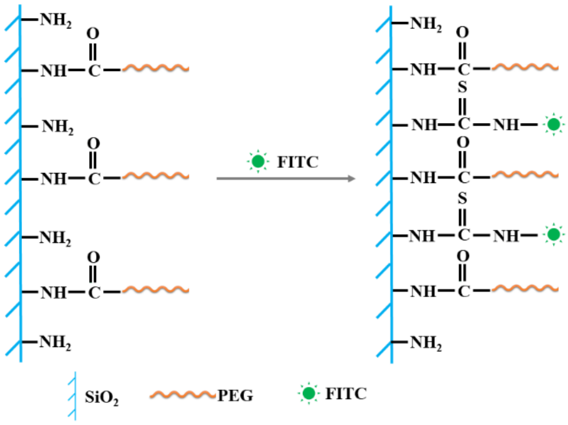

Figure S4. Schematic illustration of the surface modification with FITC. The FITC molecules were linked to the surface of nanoparticles mainly by covalent bonds (thiourea derivative).

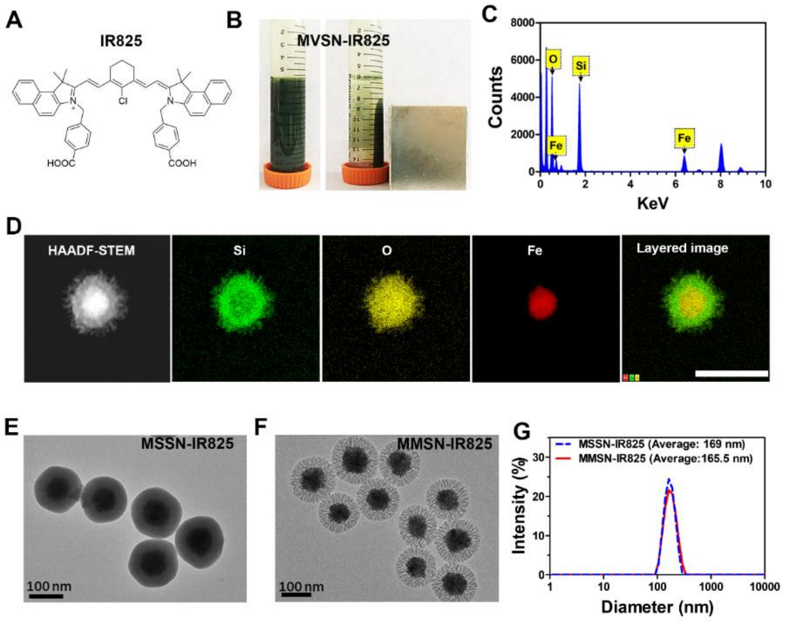

Figure S5. Characterization of nanoparticles. (A) Molecular structure of IR825. (B) Photographs of MVSN-IR825 remaining after 10 min at room temperature without (left) and with magnet (right). EDX spectrum (C) and elemental mapping (D) of MVSN (scale: 200 nm). Transmission electron microscopy (TEM) images of MSSN-IR825 (E) and MMSN-IR825 (F). (G) Size distributions of MSSN-IR825 and MMSN-IR825.

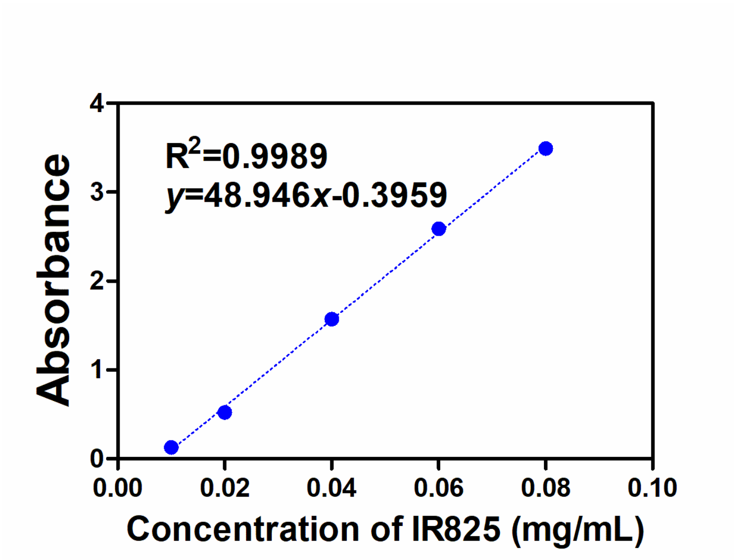

Figure S6. The concentration dependent absorbance of IR825 in ethanol.

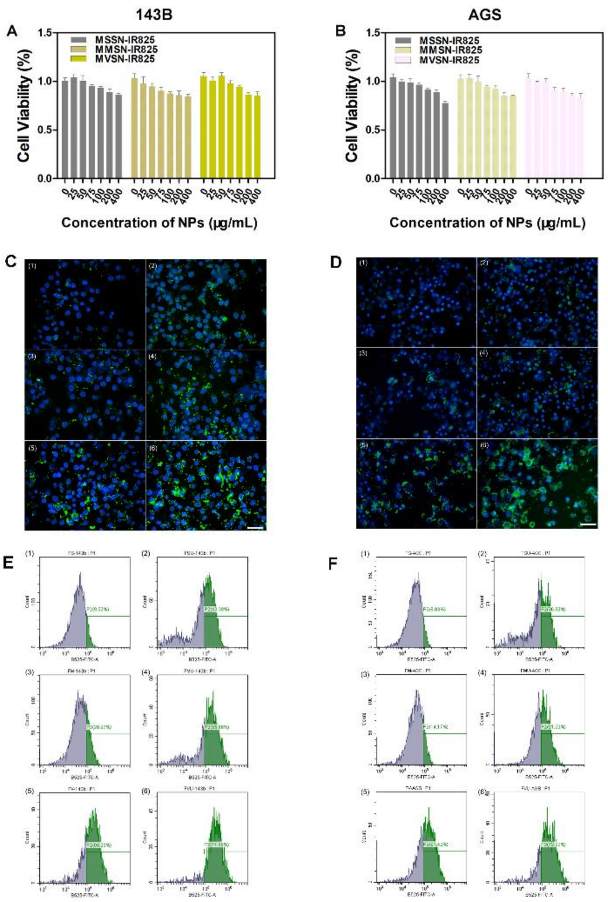

Figure S7. *In vitro* study on 143B and AGS cells endocytosis. Relative viabilities of 143B cells (A) and AGS cells (B) after 24 h incubation with FITC-labeled MSSN-IR825, MMSN-IR825 and MVSN-IR825 (n= 5 per group, mean ± SEM, two-way analysis of variance (ANOVA), Bonferroni post-test. No significant difference, $P > 0.05$). Fluorescence imaging and flow cytometry analysis for evaluating 143B (C, E) and AGS (D, F) cell phagocytosis of nanoparticles with different surface topology and with the effect of microbubble- assisted ultrasound. (1): MSSN-IR825, (2) MSSN-IR825+ MAUS, (3) MMSN-IR825, (4) MMSN-IR825+ MAUS, (5) MVSN-IR825 and (6) MVSN-IR825+ MAUS. (scale: 50 μm).

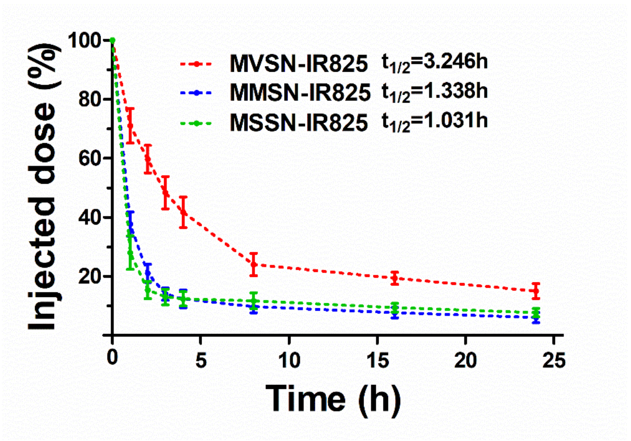

Figure S8. Percentage of the remaining injection dose of Fe element in blood after the intravenous injection of nanoparticles with three different surface topology.

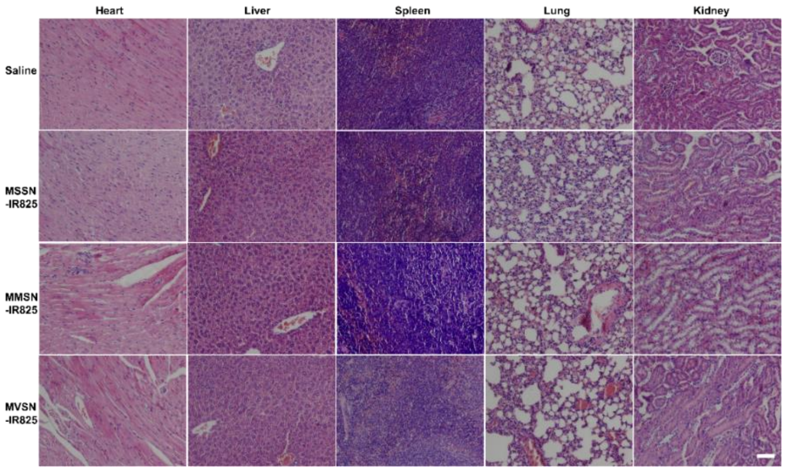

Figure S9. H&E staining of the main organs of mice in each group 24 h after the intravenous injection of all three nanoparticles (60 mg Kg^{-1}) and saline (scale: 100 μm).

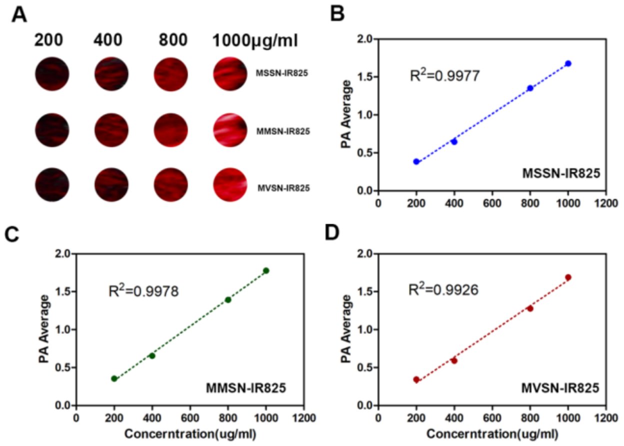

Figure S10. Photoacoustic signals (A) and the corresponding PA average value of three different surface topological nanoparticles at elevated concentrations (B, C, D).

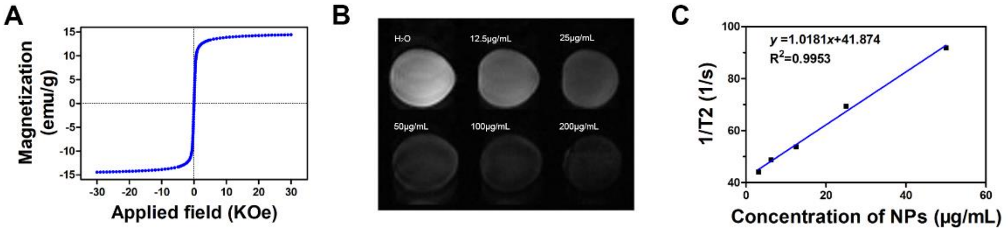

Figure S11. Magnetic properties of MVSN-IR825. (A) The hysteresis regression line of MVSN-IR825. (B) T2 imaging of MVSN-IR825 with different concentrations. (C) T2 relaxation rate of MVSN-IR825.

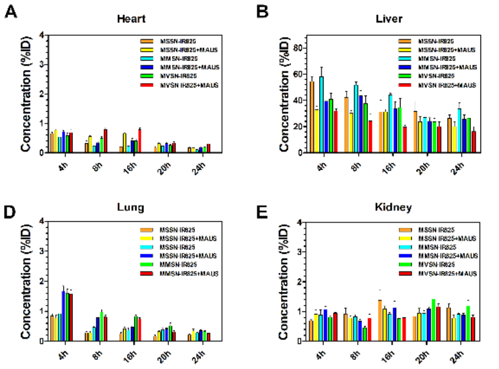

Figure S12. Tissue distribution of Fe element in tumors. (xenograft prostate cancer model). (Three mice were used for each group and each sample was measured with two repeats, mean ± SEM, two-way analysis of variance (ANOVA), Bonferroni post-test. No significant difference, P > 0.05).

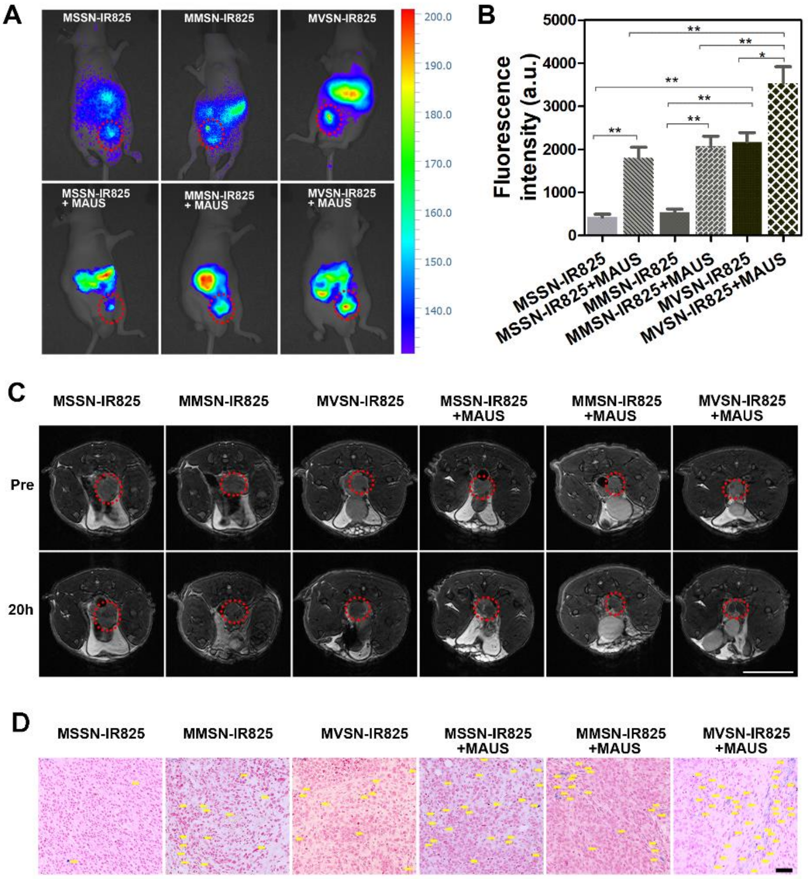

Figure S13. Imaging exhibiting the intratumor enrichment of nanoparticles in PC-3 orthotopic tumor-bearing mice. (A) *In vivo* fluorescence imaging showed intratumor enrichment of nanoparticles in the PC-3 orthotopic tumor 20 hours after intravenous administration in each group (red dotted circle represented the orthotopic tumor). (B) Quantitative analysis of fluorescence intensity per unit tumor area (n= 3 per group, mean ± SEM, one-way analysis of variance (ANOVA), Tukey post-test. *P < 0.05,

**P < 0.01). (C) MRI showing T2 signal changes of the tumor in each group before and 20 hours after administration (red dotted circle represented the orthotopic tumor). (D) Prussian blue staining showing iron distribution in PC-3 orthotopic tumor-bearing mice.

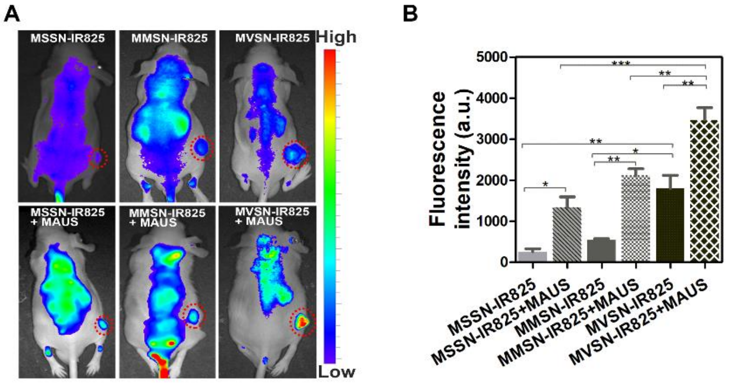

Figure S14. Imaging demonstrating the intratumor enrichment of nanoparticles 143B xenograft tumor-bearing mice. (A) *In vivo* fluorescence imaging showed intratumor enrichment of nanoparticles in tumors 20 hours after intravenous administration in each group. (B) Quantitative analysis of fluorescence intensity per unit tumor area (n= 3 per group, mean ± SEM, one-way analysis of variance (ANOVA), Tukey post-test. *P < 0.05).

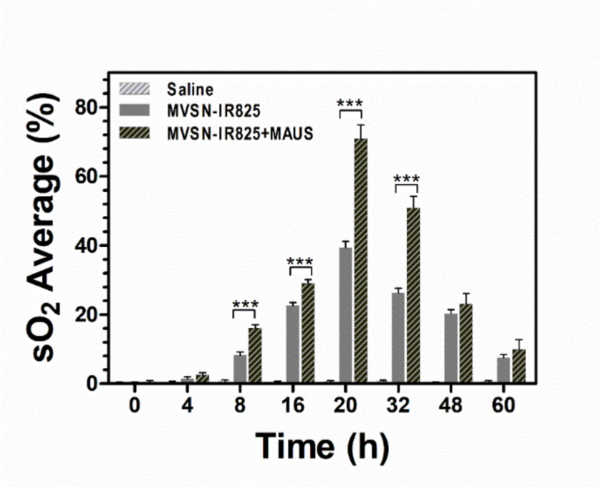

Figure S15. Quantitative data of oxygen saturation in PC-3 xenograft tumors. (n= 3 per group, mean ± SEM, two-way analysis of variance (ANOVA), Bonferroni post-test. ***P < 0.001).

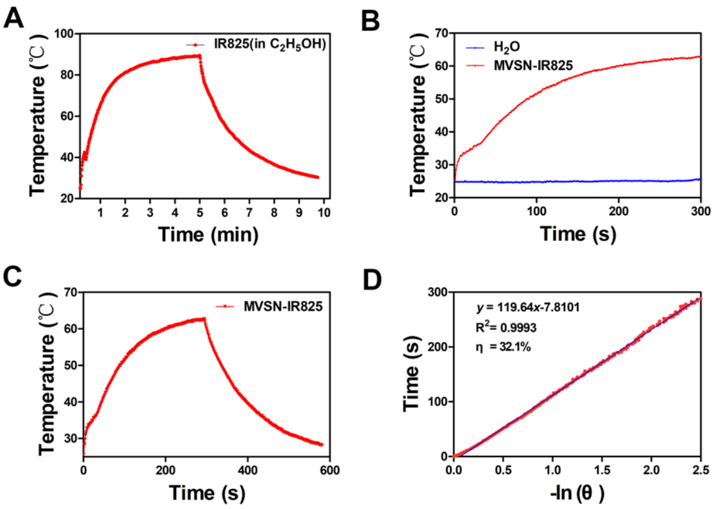

Figure S16. Photothermal performance of nanoparticles. (A) Photothermal curve of IR825 (1mg mL^{-1}) in ethanol solution. (B) Temperature changes of MVSN-IR825 (5 mg mL^{-1}) and water irradiated by laser (power density 2 W cm^{-2}). (C) Temperature changes of MVSN-IR825 (5 mg mL^{-1}) during one cycle of laser irradiation and cooling. (D) Photothermal conversion curve of MVSN-IR825.

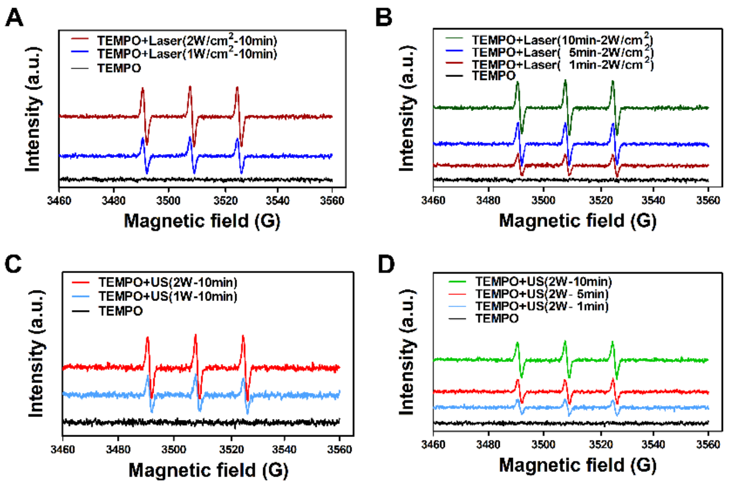

Figure S17. ESR spectrum of MVSN-IR825 (100 μg mL^{-1}) mixed with H_2O_2 (20 mM) under laser (825 nm) and low-frequency ultrasound (500 kHz) irradiation with different power and time. (A) Changes in singlet oxygen after irradiation by a near-infrared (NIR) laser (825 nm) at different powers. (B) Changes in singlet oxygen after irradiation by an NIR laser (825 nm) for different times. (C) Changes in singlet oxygen after irritation by low-frequency ultrasound (500 kHz) with different powers. (D) Changes in singlet oxygen after irradiation by low-frequency ultrasound (500 kHz) for different time period.

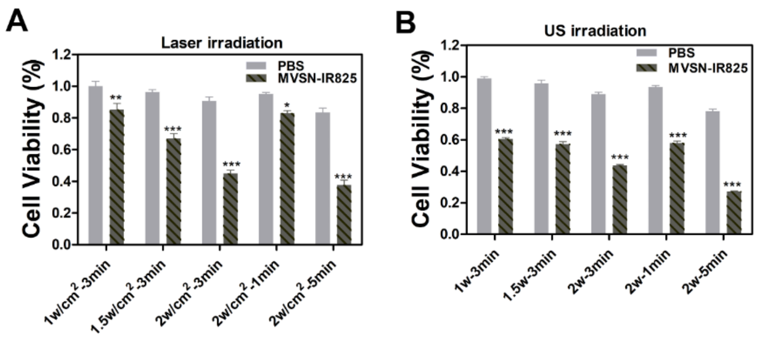

Figure S18. Cell survival rate under different treatment conditions. (A) Cell viability of PC-3 cells incubated with different laser power intensities and irradiation time (MVSN-IR825 100 μg mL^{-1}). (B) Cell viability of PC-3 cells incubated with different ultrasound power and irradiation time (MVSN-IR825 100 μg mL^{-1}). (n= 5 per group, mean ± SEM, two-way analysis of variance (ANOVA), Bonferroni post-test. In comparison to each control group. ***$P < 0.001$).

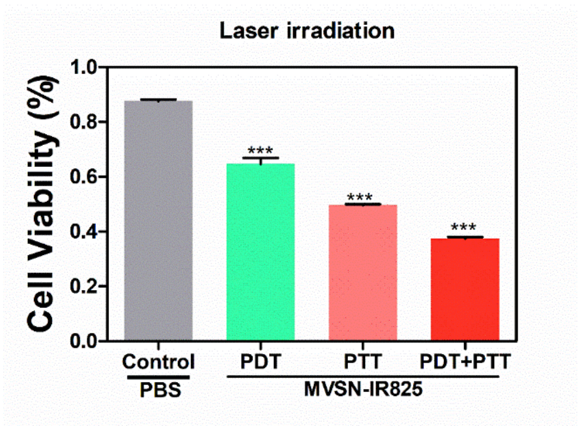

Figure S19. Survival of PC-3 cells in each group under 825 nm NIR laser irradiation (2 w cm^{-2}, 5min). PC-3 cells in control group were treated with PBS for 12h, while cells in PDT, PTT, PDT+ PTT group were incubated with MVSN-IR825 (100 μg ml^{-1}) for 12h. PDT: An ice box previously stored at -20° was placed under the 24-well plate to maintain a constant temperature during laser irradiation; PTT: treatment of cells with vitamin C (10mM). (n= 3 per group, mean ± SEM, one-way analysis of variance (ANOVA), Tukey post-test. ***$P < 0.001$).

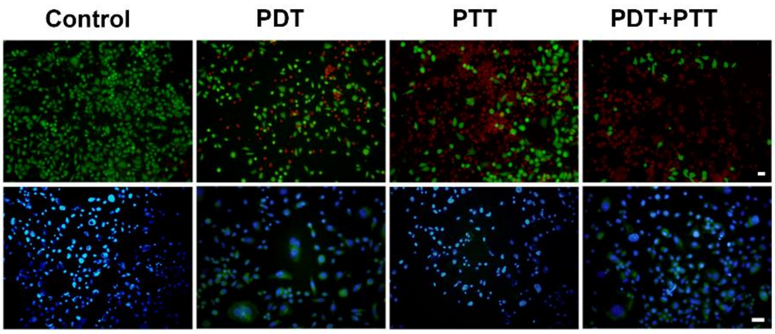

Figure S20. Fluorescence microscopy images of PC-3 cells stained with calcein-AM/PI (upper row) and DCFH-DA (lower row) after 825 nm NIR laser irradiation (2 w cm^{-2}, 5min). PC-3 cells were incubated with MVSN-IR825 (100 μg ml^{-1}) for 24h in PDT, PTT, PDT+ PTT group, and were added with PBS in control group. PDT: An ice box previously stored at -20℃ was placed under the 24-well plate to maintain a constant temperature during laser irradiation; PTT: treatment of cells with vitamin C (10mM). scale: 50 μm.

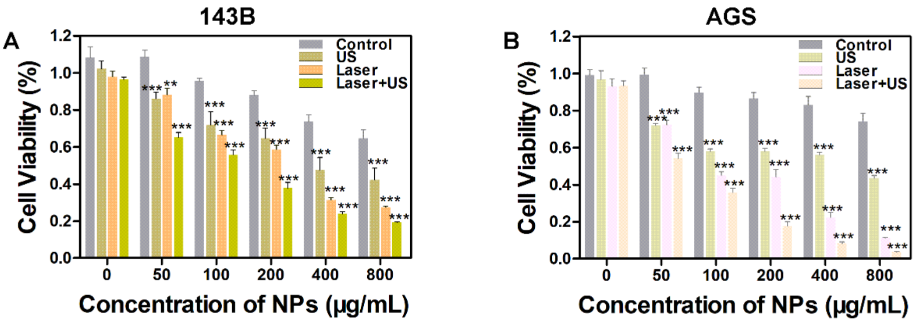

Figure S21. Cell viability 11 of 143B cells and AGS cells incubating with different concentrations of MVSN-IR825 (100 μg mL^{-1}) irradiated by laser and ultrasound (laser irradiation at 2 W cm-2 16 for 5 min, and ultrasound irradiation at 2 W for 1 min. “Laser+ US” and “US + Laser” represented different order of using the two methods; n= 5 per group, mean ± SEM, two-way analysis of variance (ANOVA), Bonferroni post-test. **P < 0.01, ***P < 0.001).

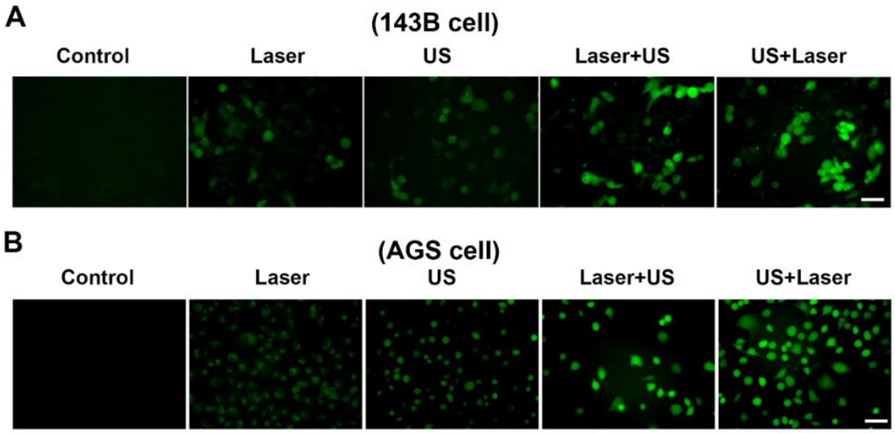

Figure S22. DCFH staining to detect the ROS produced by 143B cells (A) and AGS

cells (B) after incubation with MVSN-IR825 (100 μg mL^{-1}) in each group (laser irradiation at 2 W cm^{-2} for 5 min, and ultrasound irradiation at 2 W for 1 min. “Laser+ US” and “US + Laser” represented different order of using the two methods. scale: 50 μm).

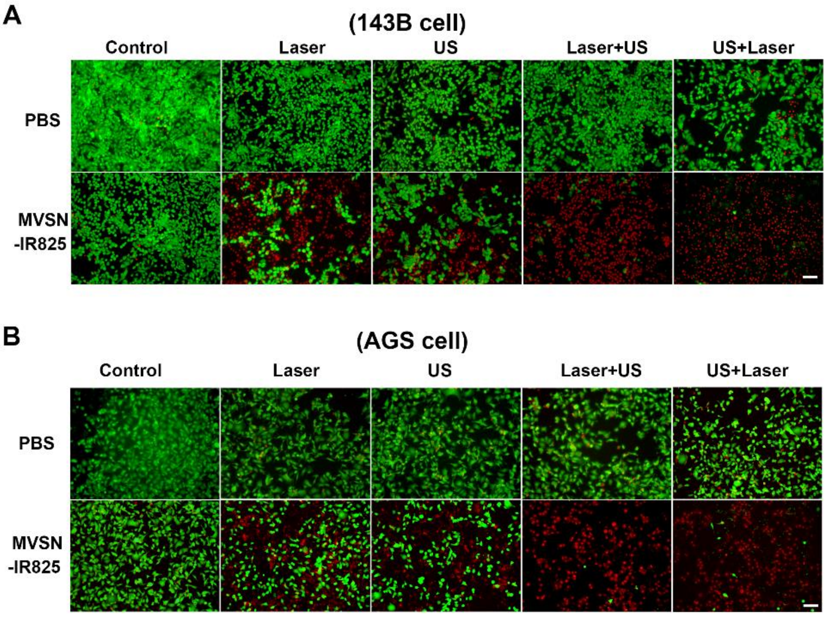

Figure S23. Calcein-AM/ PI staining of 143B (A) and AGS cells (B) incubated with MVSN-IR825 (100 μg mL^{-1}) after different treatments (laser irradiation at 2 W cm^{-2} for 5 min, and ultrasound irradiation at 2 W for 1 min. “Laser+ US” and “US+ Laser” represented different order of using the two methods. scale: 100 μm).

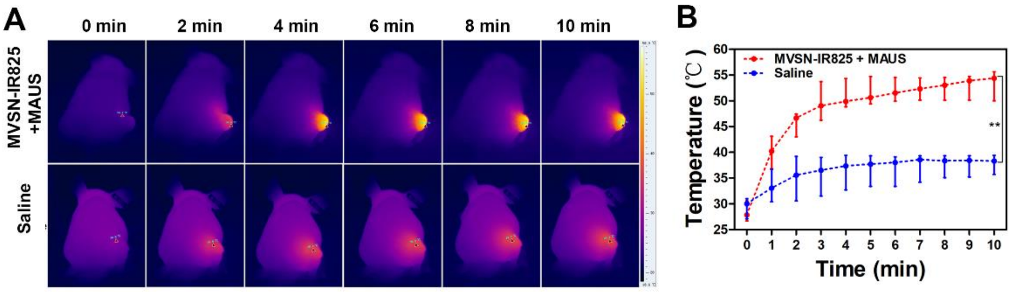

Figure S24. Infrared thermal images (A) and the temperature changes (B) of tumors in PC-3 xenograft tumor-bearing mice during laser irradiation (n= 4 per group, mean ± SEM, independent sample t-test. **P < 0.01).

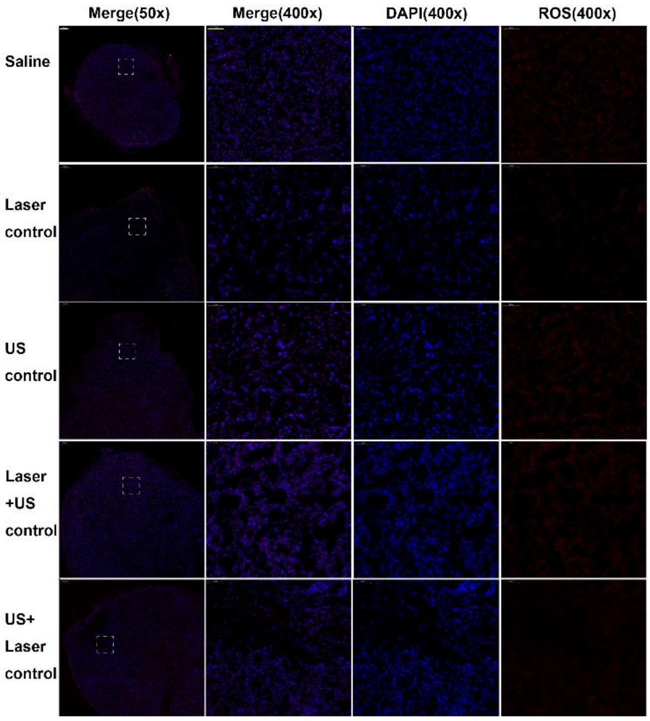

Figure S25. DHE staining of ROS of tumor tissue in PC-3 xenograft tumors (white scale is 200 μm, yellow scale is 50 μm; enlarged area inside the white dotted frame).

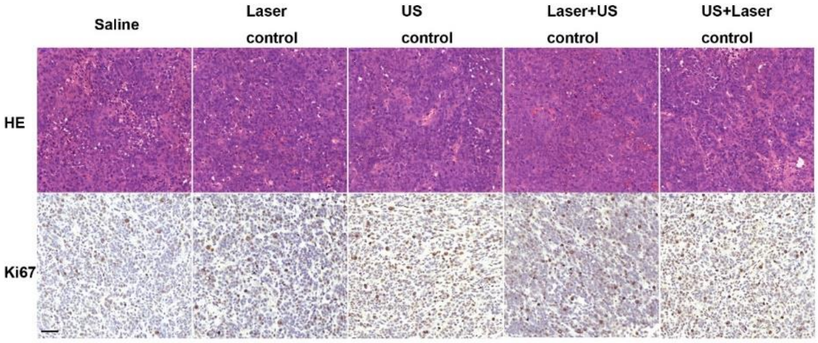

Figure S26. H&E staining and Ki67 immunohistochemical staining of tumor tissue in PC-3 xenograft tumors after treatment in each control group (scale: 50 μm).

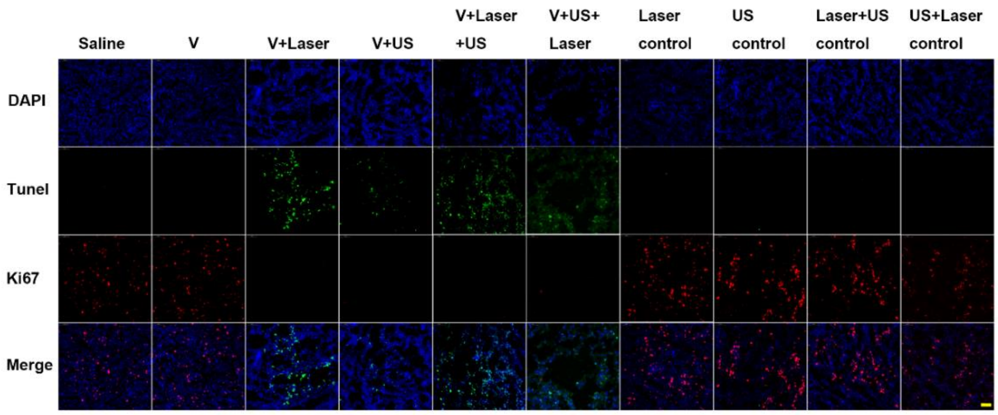

Figure S27. Immunofluorescence staining of TUNEL and Ki67 in tumor tissues in PC-3 xenograft tumors immediately after treatment in each group (scale: 50 μm).

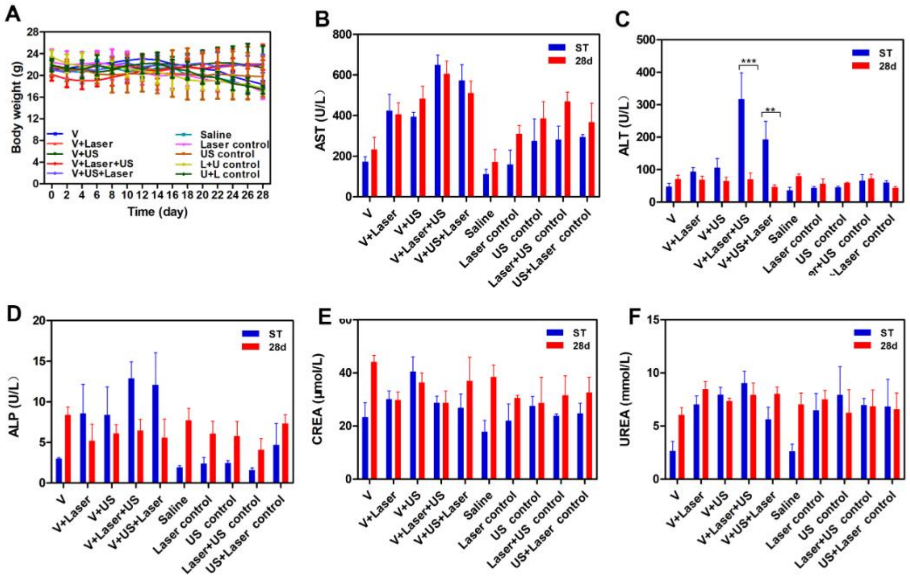

Figure S28. Evaluation of therapeutic safety in PC-3 xenograft tumor-bearing mice. (A) Changes in the body weight of mice in each group (n= 3 per group, mean ± SEM, one-way analysis of variance (ANOVA), Tukey post-test. No significant difference, P > 0.05). (B) Changes in the alanine aminotransferase (ALT) level of mice in each group. (C) Changes in the aspartic transaminase (AST) levels of mice in each group. (D) Changes in the a lkaline phosphatase (ALP) levels of mice in each group. (E) Changes in the CREA levels of mice in each group. (F) Changes in the UREA levels of mice in each group. ST: statim, immediately after treatment; 28 d: 28 days after treatment. (n= 3 per group, mean ± SEM, two-way analysis of variance (ANOVA), Bonferroni post-test. **P < 0.01, ***P < 0.001).

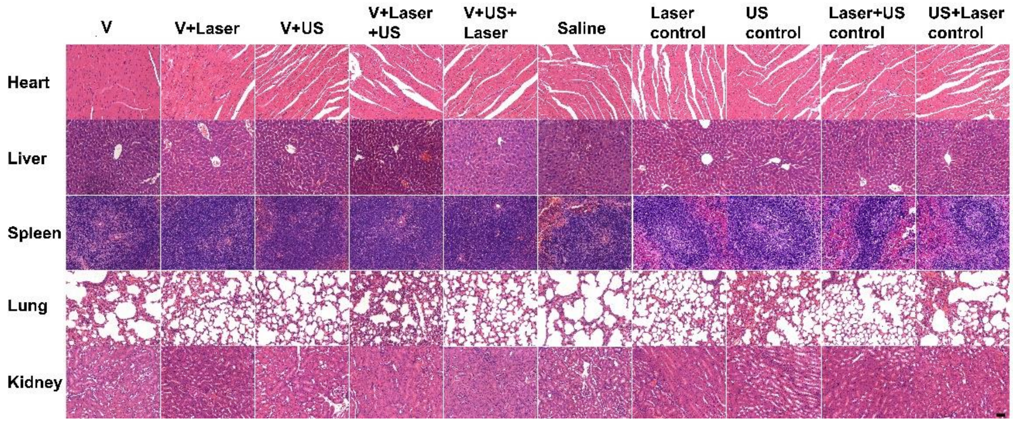

Figure S29. H&E staining of organs of PC-3 xenograft tumor-bearing mice immediately after treatment in each group (scale: 50 μm).

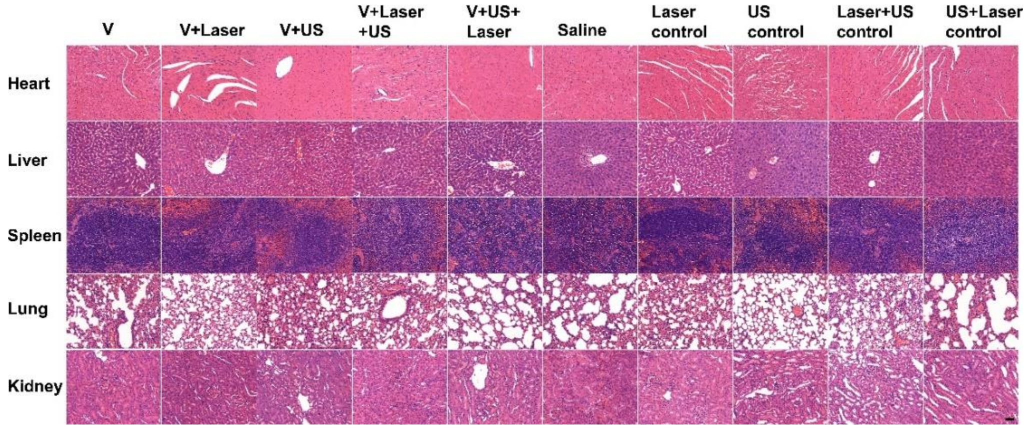

Figure S30. H&E staining of organs of PC-3 xenograft tumor-bearing mice at 28 days after treatment in each group (scale: 50 μm).

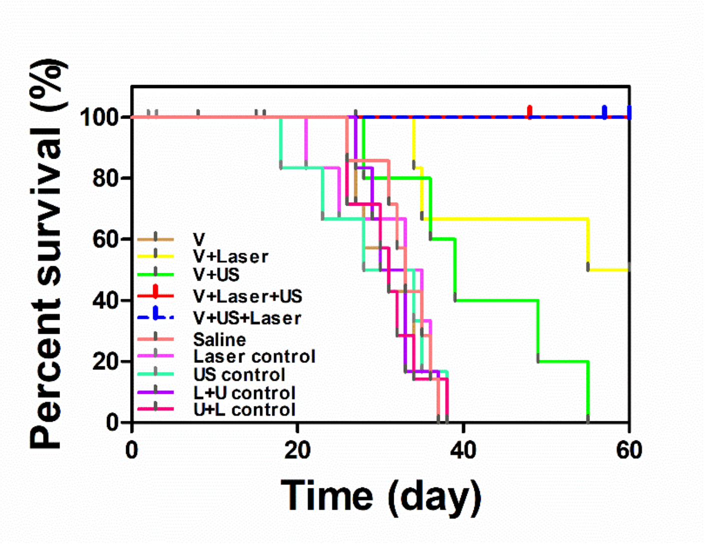

Figure S31. Percent survival of PC-3 xenograft tumor-bearing mice in all groups. The

significance of differences was evaluated by the Mantel-Cox log-rank test (n= 7 per group, mean ± SEM, In comparison to each control group. *P < 0.05, ***P < 0.001).

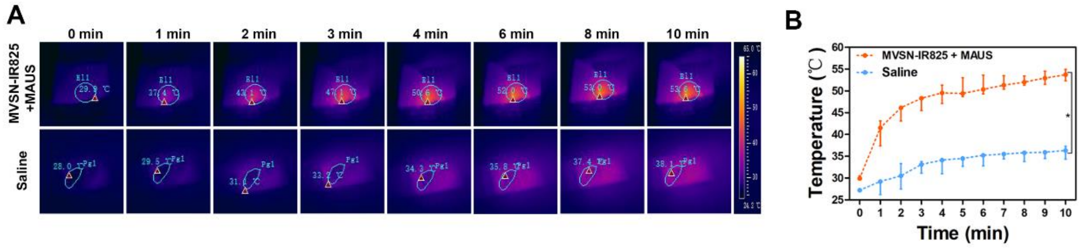

Figure S32. Infrared thermal images (A) and the temperature changes (B) of PC-3 orthotopic tumor-bearing mice during laser irradiation (n= 3 per group, mean ± SEM, independent sample t-test. *P < 0.05).

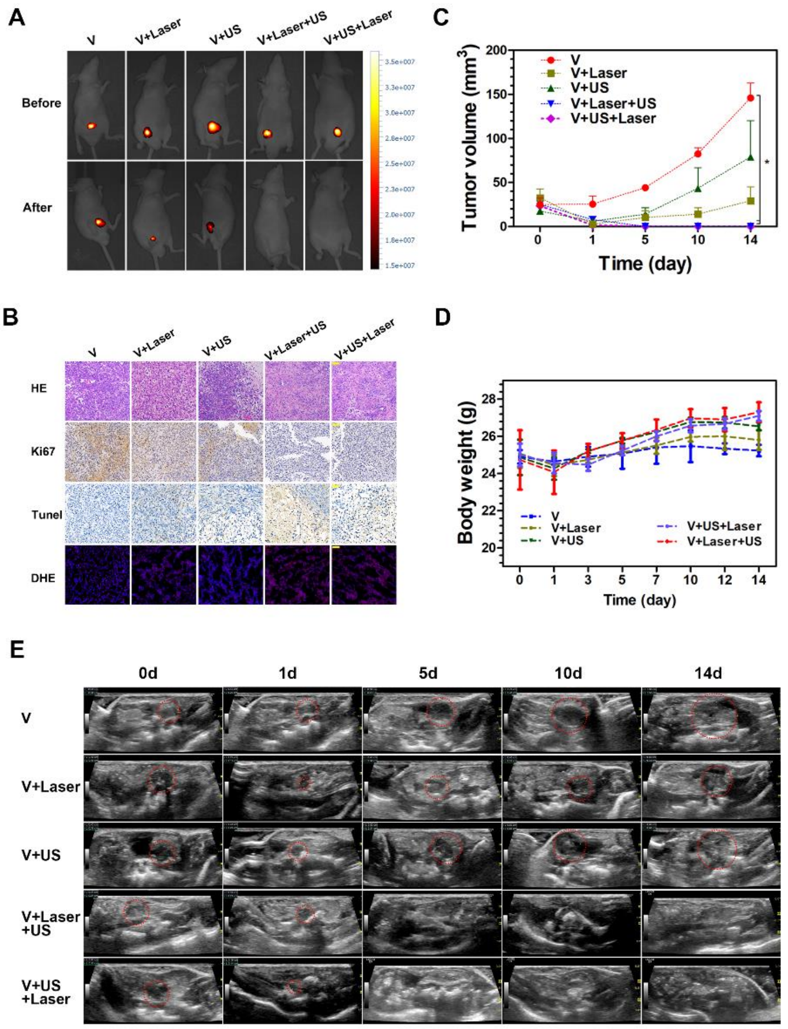

Figure S33. *In vivo* therapeutic effects of PC-3 orthotopic tumor-bearing mice after different treatments. (A) Bioluminescence monitoring the orthotopic tumor activity before and immediately after treatment. (B) H&E, Ki67, TUNEL, DHE staining of the orthotopic tumors 6 h after treatments in each group (scale: 50 μm). (C) Tumor volume changes in each group after treatment (n= 3 per group, mean ± SEM, one-way

analysis of variance (ANOVA), Tukey post-test. *P < 0.05). (D) Bodyweight changes of PC-3 orthotopic tumor-bearing mice after treatments (n= 3 per group, mean ± SEM, one-way analysis of variance (ANOVA), Tukey post-test. No significant difference, P > 0.5). (E) Ultrasound images recording the size of PC-3 orthotopic tumors after different treatments (the tumor in situ were in the red dotted circle).

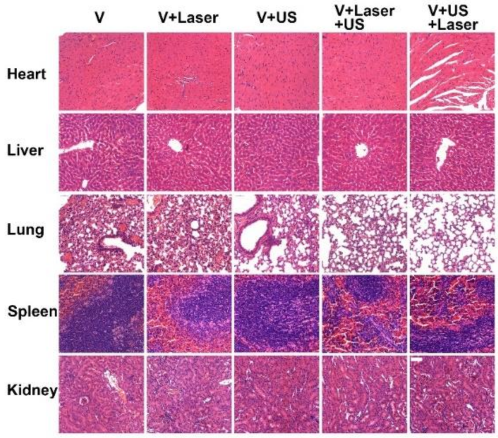

Figure S34. H&E staining of major organs of PC-3 orthotopic tumor-bearing mice 14 days after treatment in each group (scale: 50 μm).

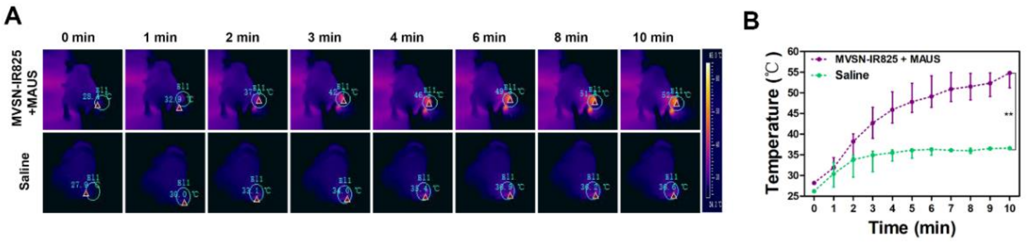

Figure S35. Infrared thermal images (A) and the temperature changes (B) of 143B xenograft tumor-bearing mice during laser irradiation (n= 3 per group, mean ± SEM, independent sample t-test. *P < 0.05).

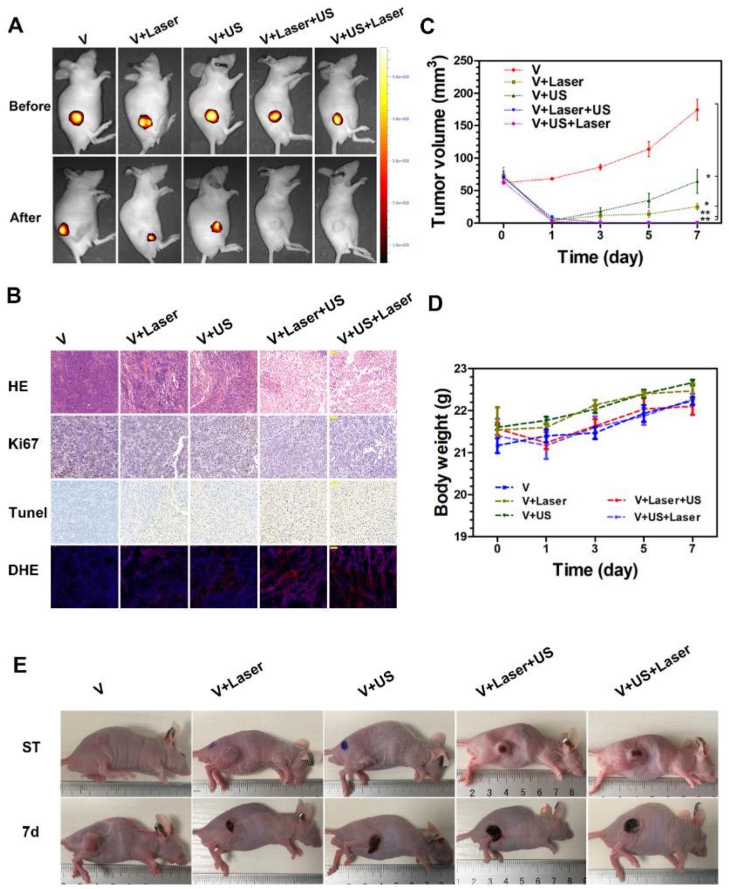

Figure S36. Monitoring therapeutic effects of 143B xenograft tumor-bearing mice. (A) Bioluminescence monitoring the 143B xenograft tumor activity before and immediately after treatment. (B) H&E, Ki67, TUNEL, DHE staining of 143B xenograft tumors 6 hours after treatments in each group (scale: 50 µm). (C) Tumor volume changes after different treatments (n= 3 per group, mean ± SEM, one-way analysis of variance (ANOVA), Tukey post-test. *P < 0.05, **P < 0.01). (D) Bodyweight changes of 143B xenograft tumor-bearing mice after treatments (n= 3 per group, mean ± SEM, one-way analysis of variance (ANOVA), Tukey post-test. No significant difference, P > 0.5). (E) Tumor morphology changes immediately and 7 days after treatments (ST: statim, immediately after treatment; 7d: 7 days after treatment).

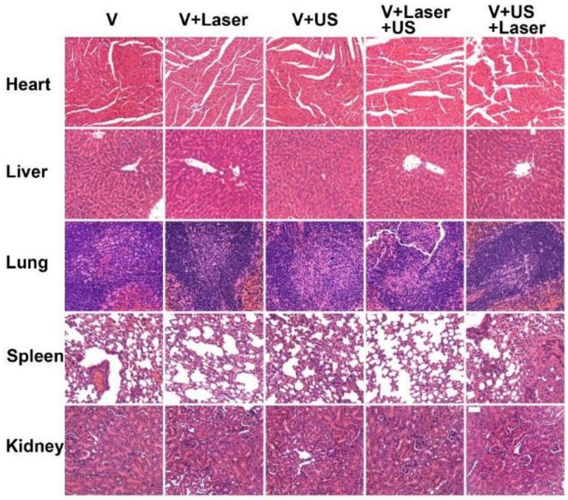

Figure S37. H&E staining of major organs of 143B xenograft tumor-bearing mice 7 days after treatment in each group (scale: 50 μm).

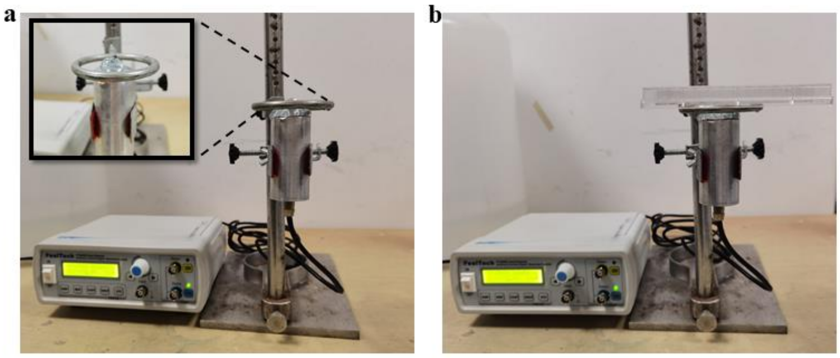

Figure S38. Details of ultrasound irradiating cells in 24-well plate. (a) the surface of the ultrasonic probe was coated with a large amount of gel, (b) the 24-well plate without cover was placed on the circular shelf while the plate bottom was in contact with the top of the gel.

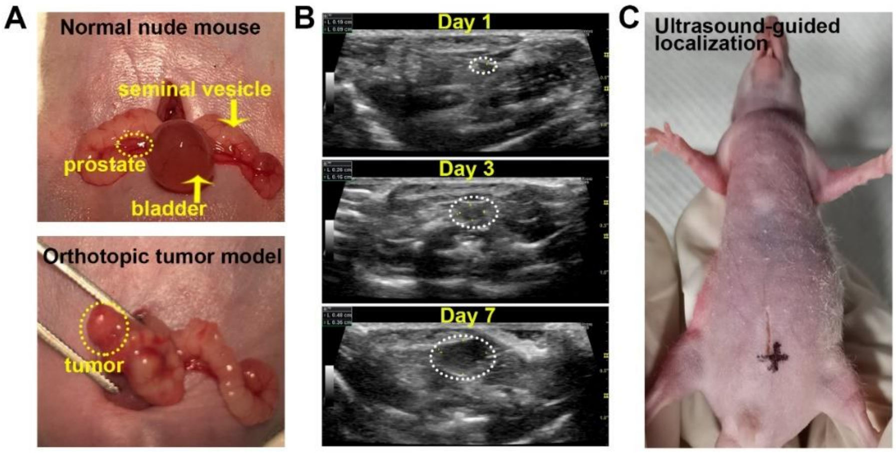

Figure S39. Construction of the PC-3 orthotopic tumor-bearing mice. (A) Topographic anatomy of normal nude mouse and the orthotopic prostate tumor model.

(B) Dynamic monitor of the tumor growth by using clinical ultrasound (The tumors were displayed in a white dotted circle, and the tumor size measured between yellow cross was shown in the upper left corner). (C) Ultrasound-guided cross localization method to display the surface projection of the orthotopic prostate tumor.

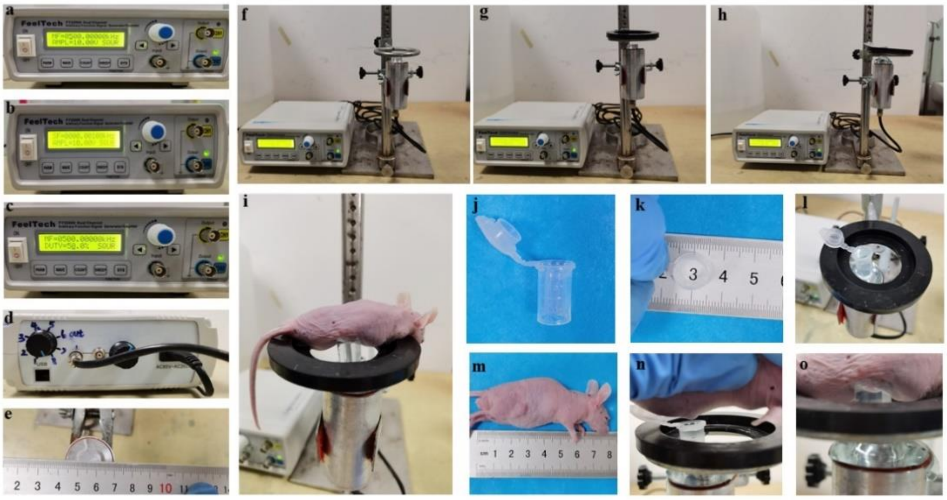

Figure S40. Details of ultrasound irradiating tumor of mice. (a)- (e) showing the details of low-frequency ultrasound instrument: (a) the transducer frequency was 500 kHz and the impulse wave was the square wave, (b) the pulse-repetition frequency was 0.001kHz, (c) the duty cycle was 50% (d) the 8 different choice for different acoustic power from 0.68 W to 2.2 W previously determined by using radiation force balances (RFB, IEC 61161: 2013), (e) the diameter of the ultrasonic probe, (f)- (i) showing the procedure of alignment with subcutaneous tumor, (j) the modified tube from cutting the 1.5 ml eppendorf tube, (k) the diameter of the modified tube, (l) the surface of the ultrasonic probe and the modified tube inside was filled with coupling gel, (m) the subcutaneous tumor model, (n) the alignment with subcutaneous tumor upon the modified tube, (o) the subcutaneous tumor was appropriately immersed into the gel.

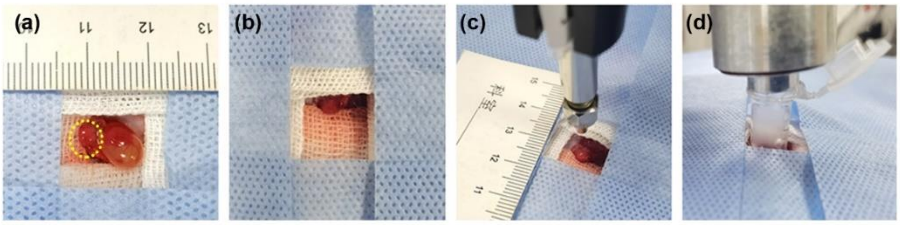

Figure S41. Therapeutic details of the PC-3 orthotopic tumors in a sterile environment. (a) Exposure the orthotopic prostate tumor, (b) protection of the surrounding organs with sterile gauze, (c) laser irradiation of the tumor site in situ, (d) ultrasound irradiation of the tumor site in situ with the assistance of a modified eppendorf tube fulling with sterile gel (the detailed information of the eppendorf tube

has been presented in Figure S40).

Discussion

Since the heart rate of mice is significantly faster than that of human beings, ultrasound contrast of subcutaneous tumors in mice can show early high perfusion within a few seconds after injection of contrast microbubbles, which demonstrates that ultrasound microbubbles can reach the tumor site fast through microcirculation. Therefore, to achieve the strongest ability of microbubble assisted ultrasound to promote nanoparticle delivery from blood vessels to tumors, nanoparticles and microbubbles were fully mixed in this study, and ultrasound irradiation of the tumor site was carried out at almost the same time as intravenous injection. The intravenous injection procedure was performed slowly for approximately 1 min, and the ultrasound irradiation time was approximately 2 min.
